# The T3SS structural and effector genes of *Chlamydia trachomatis* are expressed in distinct phenotypic cell forms

**DOI:** 10.3389/fcimb.2025.1579247

**Published:** 2025-05-08

**Authors:** Nicole A. Grieshaber, Cody Appa, Megan Ward, Alorah Grossman, Sean McCormik, Brendan S. Grieshaber, Travis Chiarelli, Hong Yang, Anders Omsland, Scott S. Grieshaber

**Affiliations:** ^1^ Department of Biological Sciences, University of Idaho, Moscow, ID, United States; ^2^ Paul G. Allen School for Global Health, Washington State University, Pullman, WA, United States

**Keywords:** *Chlamydia*, *Chlamydia* developmental cycle, bacterial differentiation, type III secretion systems, gene regulation

## Abstract

Bacteria in the Chlamydiales order are obligate intracellular parasites of eukaryotic cells. Within this order, the genus *Chlamydia* contains the causative agents of a number of clinically important infections in humans. Biovars of *Chlamydia trachomatis* are the causative agents of trachoma and the leading cause of preventable blindness worldwide, as well as sexually transmitted infections with the potential to cause pelvic inflammatory disease and infertility. Irrespective of the resulting disease, all chlamydial species share the same obligate intracellular life cycle and developmental cell forms. They are reliant on an infectious cycle consisting of at least three phenotypically distinct cell forms termed the reticulate body (RB), the intermediate body (IB), and the elementary body (EB). The EB is infectious but does not replicate. The RB replicates in the host cell but is non-infectious, while the IB is an intermediate form that transitions to the EB form. In this study, we ectopically expressed the transcriptional repressor Euo, the two nucleoid-associated proteins HctA and HctB, and the two-component sensor kinase CtcB in the RB. Transcriptional analysis using RNA-seq, differential expression clustering, and fluorescence *in situ* hybridization analysis shows that the chlamydial developmental cycle is driven by three distinct regulons corresponding to the RB, IB, or EB cell forms. Moreover, we show that the genes for the type III secretion system (T3SS) were cell type restricted, suggesting defined functional roles for the T3SS in specific cell forms.

**Importance:**

*Chlamydia trachomatis*, a sexually transmitted bacterial infection, poses a significant global health threat, causing over 100 million infections annually and leading to complications like ectopic pregnancy and infertility. This study investigates the gene expression patterns of *C. trachomatis* during its unique life cycle within human cells. As an obligate intracellular parasite, *C. trachomatis* transitions through distinct developmental stages—one for infection and dissemination, another for replication, and a third for transitioning back to the infectious form. By analyzing gene expression profiles at each stage, we identified key genes involved in these processes. Interestingly, our research also reveals the presence of two separate type III secretion system (T3SS) translocons expressed in distinct stages, suggesting their crucial roles in specific functions during the infection cycle.

## Introduction

Many bacterial species undergo dramatic phenotypic changes to adapt to different environments or to generate cells with specific physiological functions. All the bacteria in the Chlamydiales order are obligate intracellular parasites of eukaryotic cells that undergo a developmental cycle with both non-replicating and actively replicating cell forms (Rockey and Matsumoto, 2000; Abdelrahman and Belland, 2005). Chlamydial species are important pathogens of humans. *Chlamydia psittaci* causes zoonotic infections resulting in pneumonia, while *Chlamydia pneumoniae* is a human pathogen that causes respiratory disease. Different biovars of *C. trachomatis* (*Ctr*) are the causative agents of trachoma and the leading cause of preventable blindness worldwide, as well as sexually transmitted infections with the potential to cause pelvic inflammatory disease, ectopic pregnancy, and infertility (Bakken, 2007; Ohman et al., 2009; Reekie et al., 2019).

The success of a chlamydial infection depends on the completion of a complex intracellular developmental cycle consisting of multiple cell forms: the elementary body (EB), the reticulate body (RB), and the intermediate body (IB) (Rockey and Matsumoto, 2000; Abdelrahman and Belland, 2005). Although the timing of cell type conversion may differ, the broad strokes of this cycle are conserved in all the Chlamydiaceae (Schachter, 1988; Everett et al., 1999). Our current understanding of the developmental cycle as determined through promoter-reporter strains, single-inclusion kinetics, single-cell gene expression, and agent-based modeling has led to a clearer picture of the cycle (Chiarelli et al., 2020; Chiarelli et al., 2023). The EB, characterized by its condensed nucleoid and small size (~0.2-µm diameter), initiates infection of the host through the use of a type III secretion system (T3SS) and pre-formed effectors (Clifton et al., 2004; Chen et al., 2014). These effectors promote pathogen phagocytosis and entry into the targeted cell. After entry, the EB form resides in an endocytic vesicle termed the inclusion that is modified through chlamydial gene expression (Clifton et al., 2004; Chen et al., 2014). The EB completes EB-to-RB differentiation and becomes replication competent at ~10 hpi (*Ctr* serovar L2) (Miyairi et al., 2006; Abdelrahman et al., 2016). The RB, which is phenotypically characterized as larger than the EB (~1-µm diameter) and containing a dispersed nucleoid, then undergoes several rounds of amplifying replication before maturing to produce IB cells that then progress to the infectious EB, a process that takes place over ~8–10 hours after IB formation (Chiarelli et al., 2020; Chiarelli et al., 2023). The mature RBs continue to produce IB cells, acting akin to a stem cell population (Chiarelli et al., 2023). This developmental program results in a growth cycle that does not act like a typical bacterial growth culture (lag, log, and stationary phase) but instead asynchronously progresses through the RB, IB, and EB cell type transitions until cell lysis or inclusion extrusion (Chiarelli et al., 2020; Chiarelli et al., 2023).

The current understanding of the regulation of the developmental cycle comes primarily from population-level studies that frame the cycle in terms of time, treating the chlamydial population as a time-dependent uniform culture. Population-level gene expression data have been determined for chlamydial infections and have been described according to time after infection. These studies include RT-qPCR, microarray, and RNA-seq studies and contribute to the canonical early (~0–10 hpi, EB-to-RB differentiation), mid-cycle (~10–18 hpi, RB replication), and late (~18-hpi cell lysis, EB formation) gene expression paradigm (Shaw et al., 2000; Nicholson et al., 2001; Belland et al., 2003; Wurihan et al., 2021; Wurihan et al., 2024). The reliance on population-level data from this mixed-cell population has confounded the understanding of gene expression as it pertains to the specific chlamydial cell forms.

Here, we sought to define the transcript profiles of the cell forms that underpin the observed growth cycle by investigating the effects of the ectopic expression of four transcriptional regulatory proteins in *C. trachomatis* (*Ctr*): Euo, HctA, HctB, and CtcB. Euo (Early Upstream Open Reading Frame (ORF)) is among the earliest genes expressed post EB-to-RB differentiation during chlamydial infection (Hakiem et al., 2023; Rosario et al., 2014; Zhang et al.,1998). Current evidence suggests that Euo is a DNA-binding protein that acts to repress a handful of late-cycle genes (Hakiem et al., 2023; Rosario et al., 2014; Zhang et al., 1998), and Euo ectopic expression leads to a block in the developmental cycle (Appa Cody et al., 2024). HctA is a small DNA-binding protein with limited homology to the histone H1 histone family and is expressed transiently in the IB cell type ~8–10 hours before HctB (Barry et al., 1990; Chiarelli et al., 2020; Chiarelli et al., 2023). HctA has been shown to bind DNA and to repress transcription broadly across chromosomes of both *Ctr* and, when ectopically expressed, *Escherichia coli* (Grieshaber et al., 2006; Barry et al., 1990; Grieshaber et al., 2004). HctB is a second small positively charged protein that has limited homology to the H1 histone family and is thought to contribute to the condensation of the EB nucleoid (Brickman et al., 1991). Our data show that, unlike HctA, HctB is expressed late in EB development, during the final stages of EB formation (Chiarelli et al., 2020; Chiarelli et al., 2023). In addition to these DNA-binding proteins, *Ctr* contains a single cytosolic two-component regulatory system (TCS) consisting of CtcB/CtcC (histidine kinase/response regulator) (Koo et al., 2006; Koo and Stephens, 2003). The *Ctr* TCS is actively transcribed during RB-to-EB development, and the protein products are functional with respect to phosphotransfer (Koo and Stephens, 2003). Additionally, the expression of the ATPase effector domain of the response regulator, CtcC, resulted in the upregulation of the sigma54 regulon, which included many developmentally regulated genes (Soules et al., 2020). We expect that the ectopic expression of CtcB would phosphorylate CtcC and amplify the signaling and activation process of the sigma54 gene expression.

We have shown that Euo, HctA, and HctB promoter activities help define the RB, IB, and EB cell forms (Chiarelli et al., 2020; Chiarelli et al., 2023). Therefore, along with CtcB, we determined the effects of the ectopic expression of these regulatory factors on the transcriptome of *Ctr* using RNA-seq. Our data produced gene regulation profiles consistent with cell form-specific transcriptomes. This allowed us to assign/predict the expression of a large fraction of the chlamydial genome into RB-, IB-, and EB-specific transcript categories. Within our cell form expression prediction groups were a number of T3SS genes. Using fluorescence *in situ* hybridization (FISH) in the context of cell type promoters, we showed that components of the T3SS were expressed in specific cell forms.

## Results

### Ectopic expression of Euo, HctA, CtcB, and HctB resulted in arrest of the developmental cycle

To determine the effects of the ectopic expression of Euo, HctA, HctB, and CtcB on gene expression and the developmental cycle, we expressed these proteins as well as the GFP protein Clover (control) under the control of the T5 promoter and theophylline-responsive riboswitch from the native chlamydial plasmid (Grieshaber et al., 2022). We infected cells with the strains L2-E-euo-FLAG, L2-E-hctA-FLAG, L2-E-ctcB-FLAG, L2-E-clover-FLAG, and L2-tet-J-E-hctB-FLAG, and we induced protein expression at 15 hpi. We chose to induce expression at 15 hpi in order to evaluate the effects of these proteins on the RB-to-EB stage of the developmental cycle. At 15 hpi, the vast majority of the chlamydial cells would be in the RB form (Chiarelli et al., 2023). A single band of the appropriate size was visible by Western blotting for the induced samples, while no band was visible in the uninduced samples for all constructs (**Supplementary Figure S1**).

**Figure 1:**
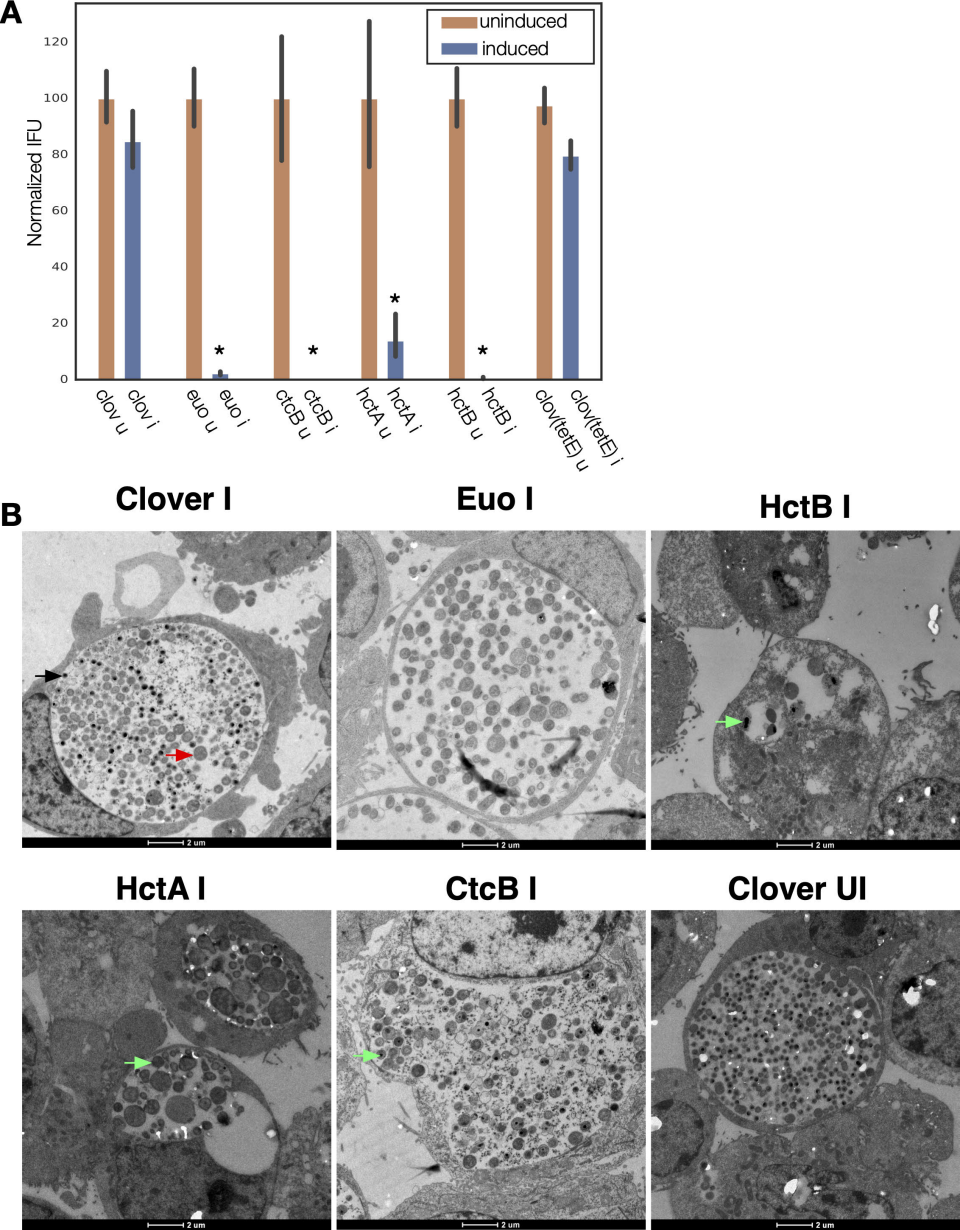
Ectopic expression of Euo, HctA, CtcB, and HctB resulted in inhibition of the developmental cycle. **(A)** Cell monolayers were infected with the four strains and the Clover control strains and induced ectopic expression at 15 hpi. EBs were harvested at 48 hpi. IFU production was dramatically reduced by the ectopic expression of Euo, HctA, CtcB, and HctB but not by the expression of the Clover protein. *p < 0.01. **(B)** Transmission EM of Cos-7 cells infected with *Ctr* expressing Clover, Euo, HctB, HctA, or CtcB. Ectopic expression was induced at 15 hpi, and the cells were fixed and prepared for imaging at 30 hpi. The bacteria in the induced (I) and uninduced (UI) Clover control chlamydial infections looked similar with inclusions of both samples containing large RB-like cells (red arrow) as well as electron-dense EB-like cells (black arrow). The *Ctr* in the Euo-expressing inclusions were primarily RB-like cells, while very few cells were electron-dense EB cells. The chlamydial cells in the HctB expressing inclusions were abnormal looking, some with apparent condensed nucleoids (green arrows). The HctA-expressing *Ctr* also appeared abnormal with condensed nucleoids. Many of the CtcB-expressing *Ctr* cells were target-like RB-sized cells with a condensed nucleoid (green arrows). EBs, elementary bodies; EM, electron microscopy; RB, reticulate body.

The production of infectious progeny (EBs) was tested using a reinfection assay at 48 hpi. The ectopic expression of all four transcriptional regulatory proteins resulted in a significant inhibition of EB production as compared to the Clover-FLAG controls (**Figure 1A**). In addition, infected cells were imaged using transmission electron microscopy (TEM). For TEM, cells were infected with the four strains plus the Clover control strains, induced for expression at 15 hpi, and fixed and prepared for TEM analysis at 30 hpi (**Figure 1B**). The induced and uninduced Clover-FLAG control samples were indistinguishable. The inclusions for both samples had similar ratios of RBs (large cell forms) and EBs (small electron-dense forms). Cells ectopically expressing Euo had inclusions with very few visible EBs (small electron-dense forms), and most cells appeared RB-like (large less electron-dense cell forms). The inclusions for the HctB- and HctA-expressing bacteria contained small populations of abnormal RB-like forms as well as cells with dense structures resembling condensed nucleoids (electron-dense regions inside cells). The inclusions of the CtcB-expressing bacteria contained both RB- and EB-like cells and an increase in intermediate forms, i.e., RB-sized cells with condensed nucleoids (**Figure 1B**). These data suggested that the ectopic expression of all four of these transcriptional regulatory proteins resulted in an aborted developmental cycle as indicated by both the inclusion forming units (IFU) measurements and the dysregulated cell forms seen by EM.

#### RNA-seq of the ectopically expressing chlamydial strains

To better understand the effects of the ectopic expression of each regulatory protein, we used RNA-seq to characterize the corresponding transcriptomes. We infected host cells with each strain (L2-E-clover-FLAG, L2-E-euo-FLAG, L2-E-hctA-FLAG, L2-E-ctcB-FLAG, L2-tet-J-E-hctB-FLAG, and L2-tet-J-E-clover-FLAG), induced expression at 15 hpi, and harvested RNA for library construction at 18 and 24 hpi. We chose to investigate gene expression 3 hours after induction (18 hpi) to capture potential immediate effects on the developmental cycle. We also investigated gene expression at 9 hours after induction (24 hpi). This later time point allowed for the detection of changes between the advancement of the cycle in control samples and the potential inhibition of the cycle by the ectopic expression of the regulatory proteins.

**Figure 2:**
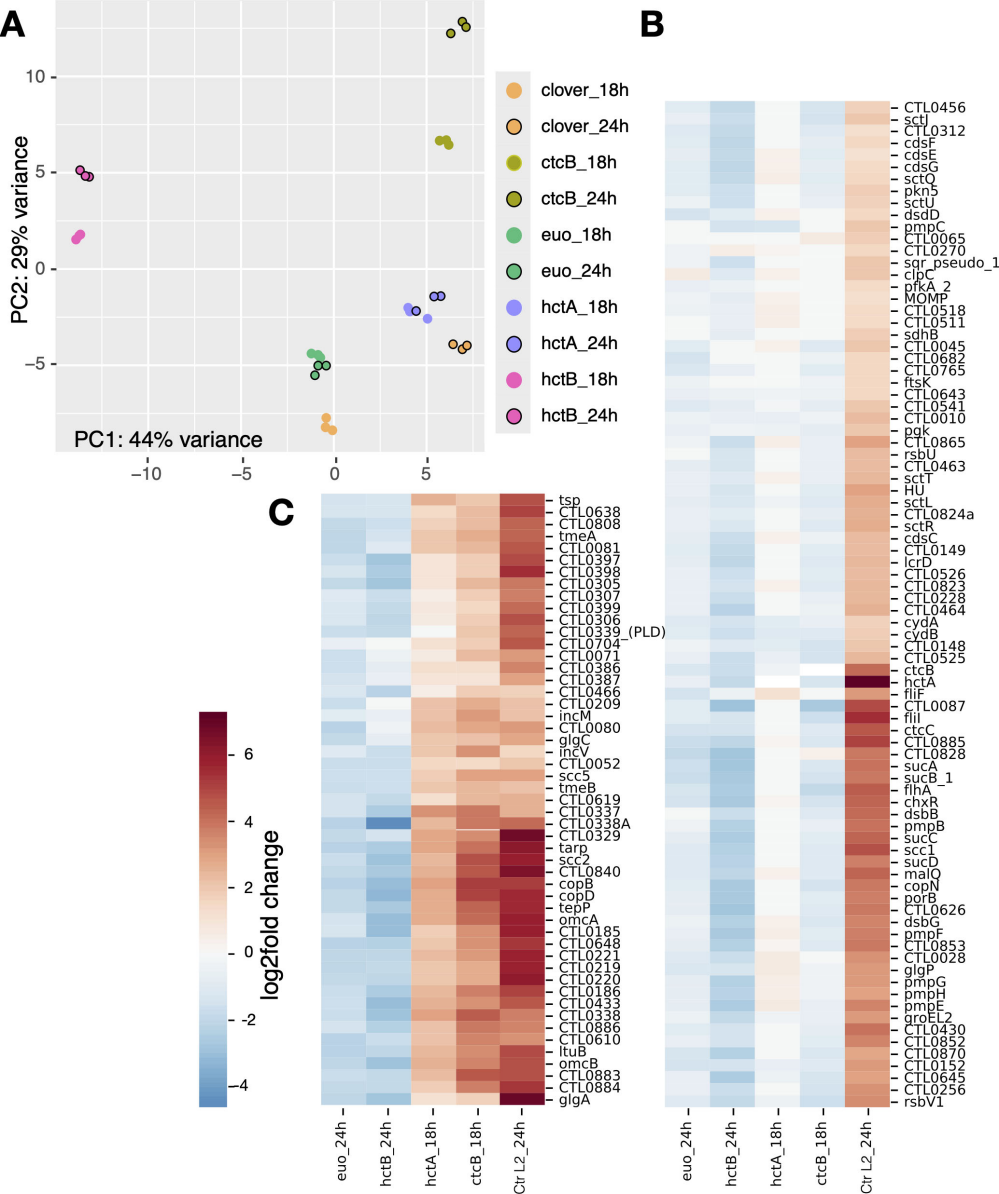
RNA-seq analysis of *Ctr* ectopically expressing Euo, HctA, HctB, or CtcB. **(A)** For each of the induced samples (n = 3), the RNA-seq PCA profiles clustered within the same ectopic expression group, but each group had a distinct profile as visualized by plotting the first and second principal components. **(B, C)** Hierarchically clustered heatmap plots revealed two distinct late gene regulation groups. **(B)** The IB cluster group was defined as genes that were upregulated between wt *Ctr* (*Ctr* L2_24h) infections at 18 and 24 hpi (late genes) but were not upregulated by the ectopic expression of HctA and CtcB as compared to the Clover control. **(C)** The EB gene cluster was defined as genes that were upregulated between wt *Ctr* (*Ctr* L2_24h) infections at 18 and 24 hpi (late genes) and were upregulated by ectopic expression of HctA and CtcB as compared to the Clover control. PCA, principal component analysis; IB, intermediate body; EB, elementary body.

We compared the transcriptome of each sample in triplicate using principal component analysis (PCA). As expected, each set of triplicate biological replicates clustered closely together (**Figure 2A**). The Clover control samples clustered in distinct groups depending on isolation time point (i.e., 18 vs. 24 hpi) (**Figure 2A**). For the Euo-expressing samples, all the 18- and 24-hpi samples clustered closely together, suggesting only small differences in gene expression between the time point samples. This was also seen for the HctA expression; the 18- and 24-hpi experimental samples clustered closely together, again suggesting only small differences between time point samples (**Figure 2A**). For the HctB 18- and 24-hpi experimental samples, each time point clustered separately, but the two clusters were closer to each other than to any of the other experimental conditions. The CtcB 18- and 24-hpi experimental samples were similar, the replicates for each time point clustered tightly together, and the 18- and 24-hpi samples clustered closer to each other than to the samples from the other experimental conditions (**Figure 2A**). Together, these data suggest that each ectopically expressed protein generated a unique gene expression pattern.

We compared RNA-seq data sets from each induced ectopic expression experiment to the induced Clover controls, 18-hpi Clover to 18-hpi experimental sample, and 24-hpi Clover to 24-hpi experimental samples. In addition to data from the current analysis, we used the gene expression data from our previously published time course data set (Omsland et al., 2012). From this data set, we determined the differential gene expression between wt *Chlamydia* from the 18-hpi sample and the 24-hpi sample (*Ctr* L2_24h, **Figures 2B, C**), capturing changes in late gene expression (RB to IB and EB). We compared this differential gene expression pattern to the differential gene expression patterns of the ectopic expression experimental data. We generated a hierarchically clustered heatmap using the Seaborn clustering algorithm (Waskom, 2021) (**Figures 2B, C**). We used the 24-hpi samples for Euo and HctB for clustering analysis, as these proteins acted as inhibitors and blocked cycle progression (**Figures 2B, C**). The 24-hpi samples allowed more time for accumulated changes as the Clover controls progressed to the production of late genes, while the Euo- and HctB-expressing samples did not. We used the 18-hpi data from the HctA and CtcB ectopic expression experiments for cluster analysis, as they both acted as inducers (**Figures 2B, C**). These changes were the most obvious in the 18-hpi samples, as the Clover controls had yet to express late genes.

Clustering produced two dominant groups (**Figures 2B, C**). Both groups featured genes that were dramatically upregulated between 18 and 24 hpi during the wt infection (**Figures 2B, C**, *Ctr* L2_24h), suggesting that all of these genes would be considered late genes (Shaw et al., 2000; Belland et al., 2003). The major difference between the two cluster groups was the changes in gene expression induced by HctA and CtcB ectopic expression (**Figures 2B, C**). One of the clusters featured genes that were not dramatically induced upon HctA-FLAG or CtcB-FLAG ectopic expression (**Figure 2B**). The other cluster showed the opposite with all the genes upregulated by the ectopic expression of HctA-FLAG or CtcB-FLAG (**Figure 2C**). The ectopic expression of Euo-FLAG and HctB-FLAG led to a downregulation of both sets of genes as compared to the Clover control (**Figures 2B, C**). Many of the genes in the first cluster (**Figure 2B**) have been shown to be expressed mid-cycle or late cycle (Shaw et al., 2000; Belland et al., 2003), while most of the genes in the second cluster (**Figure 2C**) have been identified to be expressed late in the developmental cycle (Shaw et al., 2000; Belland et al., 2003). Additionally, many of the second cluster genes have been identified as sigma54/ctcB-ctcC-regulated genes (Soules et al., 2020; Hatch Nathan and Ouellette Scot, 2023). These data suggest that the late-expressed genes can be divided into two distinct categories: those expressed in the IB (**Figure 2B**) and those expressed in the infectious EB (**Figure 2C**).

#### Gene expression cluster groups map to cell type-specific gene expression profiles

Using the clustering data observations, we created selection criteria to categorize the RNA-seq data into three gene expression groups (**Supplementary Table S1**). The first group was genes for which we observed little to no change after Euo ectopic expression when compared to the Clover control and had little to no change in gene expression between 18 and 24 hpi during infection with wt Ctr. We separated the late genes into two groups. We defined the first group as genes whose expression increased between 18 and 24 hpi in the wt infection but were not induced by the HctA, CtcB, or Euo ectopic expression. We defined the second group as genes whose expression was increased from 18 to 24 hpi in the wt infection and were upregulated by the CtcB and HctA ectopic expression but not increased by Euo ectopic expression. Based on the observation that *euo* was a member of the first group, we defined these genes as RB genes (**Supplementary Table S1**). For the two late gene groups, we noticed that *hctA*, an IB gene (Chiarelli et al., 2020; Chiarelli et al., 2023), was a member of the first group and therefore designated this group as IB genes (**Supplementary Table S1**). We designated the second late gene group as EB genes, i.e., the regulon likely involved in the final stage of generating infectious EBs. This group contains the *hctB*, *tarp*, and *scc2* genes, which we have previously shown to be expressed very late in the IB-to-EB developmental progression (Chiarelli et al., 2020; Chiarelli et al., 2023).

**Figure 3:**
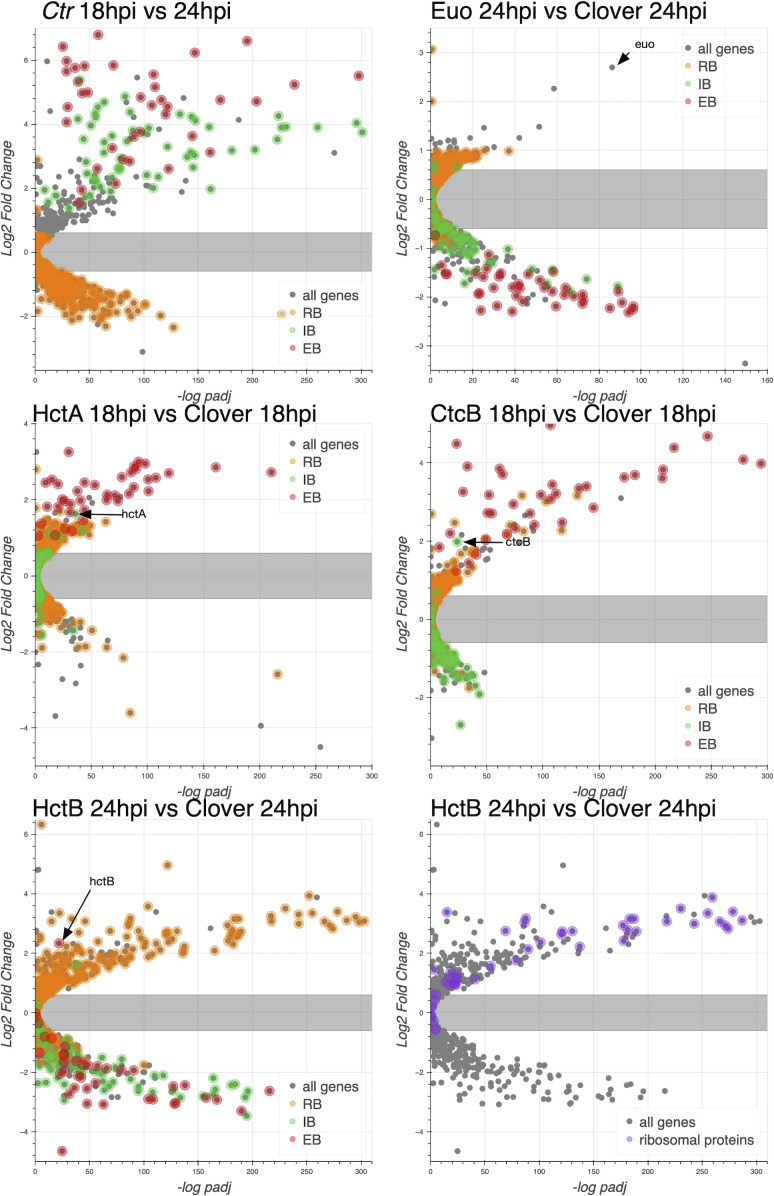
Effects of ectopic expression of Euo, HctA, CtcB, and HctB on the gene expression of every *Ctr* gene. RNA-seq differential expression was determined for each gene comparing wt infection at 18 hpi vs. 24 hpi, Euo-FLAG expression, HctA-FLAG expression, CtcB-FLAG expression, and HctB-FLAG expression vs. the control Clover-FLAG. For the wt infection, volcano plots show that the expression of RB-designated genes (orange) was largely unchanged from 18 to 24 hpi, while IB- (green) and EB-designated (red) genes were dramatically upregulated. For Euo-FLAG expression experiment, the RB genes (orange) were largely unchanged, while IB genes (green) and EB genes (red) were all downregulated. Ectopic expression of HctA-FLAG resulted in the repression of many of the RB genes (orange) and upregulation of the EB genes (red) but had little impact on the expression of the IB genes (green). CtcB-FLAG expression had very little effect on RB genes (orange) but dramatically upregulated EB genes (red). The ectopic expression of HctB-FLAG resulted in the downregulation of both IB (green) and EB (red) genes but upregulated many RB genes (orange). Additionally, HctB-FLAG expression resulted in the upregulation of many of the ribosomal protein genes (purple). RB, reticulate body; IB, intermediate body; EB, elementary body.

We next used volcano plots to visualize the individual effects of each of the ectopic expression constructs on changes in gene expression of all *Ctr* genes. We plotted the expression changes (log2fold change) vs. statistical significance (−log of the p-value), and we highlighted the RB, IB, and EB gene groups listed in **Supplementary Table S1** (**Figure 3**). We plotted changes in gene expression from 18 to 24 hpi from a wt infection, which as expected indicated that the genes from **Supplementary Table S1** designated as RB genes were largely unchanged in gene expression between 18 and 24 hpi, while both the designated IB and EB genes showed increased expression. RB gene expression was for the most part unchanged when Euo was ectopically expressed, in contrast to dramatic reductions in the expression of both IB- and EB-designated genes (**Figure 3**, Euo 24hpi vs. Clover 24hpi). The ectopic expression of both HctA and CtcB dramatically increased the expression of EB genes (**Figure 3**, HctA and CtcB 18hpi vs. Clover 18hpi). HctB ectopic expression resulted in dramatic repression in the expression of both IB and EB genes while increasing the expression of a subset of RB genes relative to Clover (**Figure 3**, HctB 24hpi vs. Clover 24hpiE). We noticed that many of the genes that showed increased expression when HctB was ectopically expressed were ribosomal protein genes (**Figure 3**, HctB 24hpi vs. Clover 24hpi).

#### Verification of cell type-specific gene expression by fluorescence *in situ* hybridization

**Figure 4:**
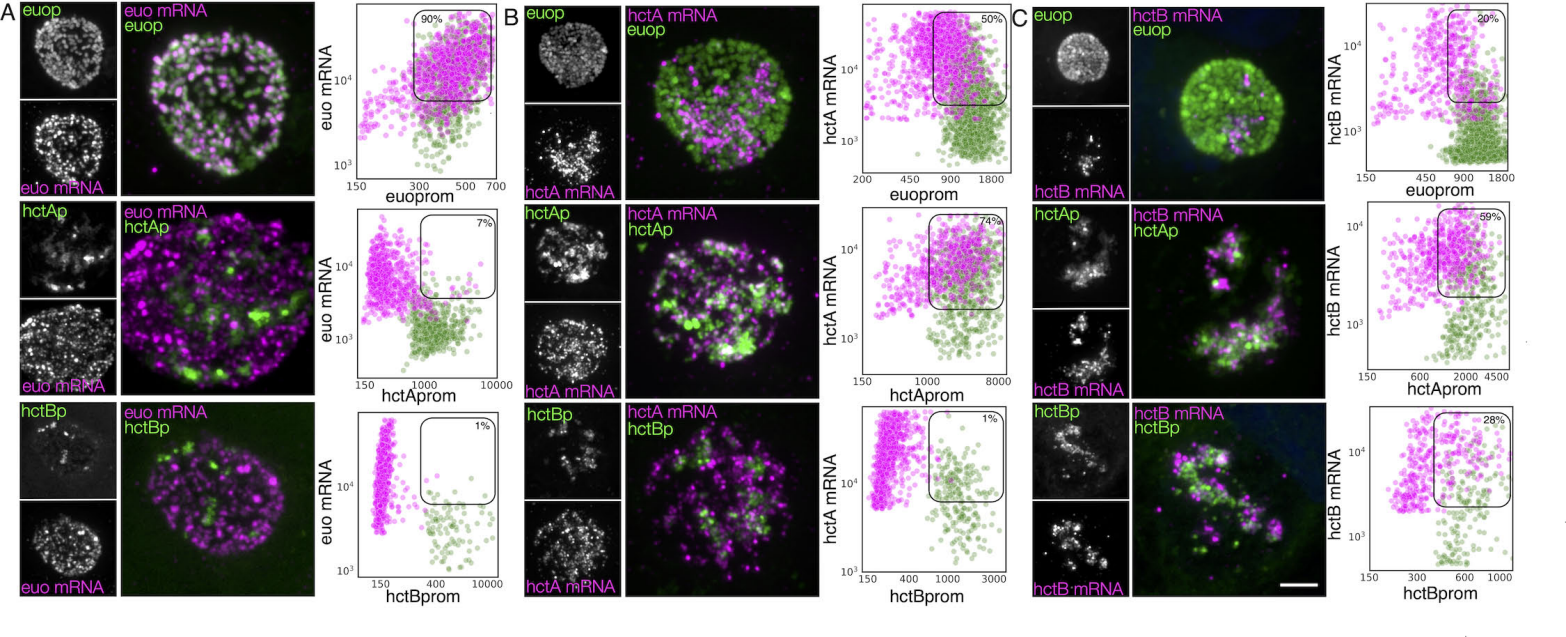
Cell type expression of representative genes from the three gene categories (RB, IB, and EB) correspond to the three chlamydial cell forms. Cos-7 cells infected with L2-AsciEng or L2-BsciEng, fixed at 24 hpi, and stained using FISH probes for *euo* mRNA, *hctA* mRNA, and *hctB* mRNA. **(A)** Z-projection confocal micrographs showing *euo* mRNA localization in comparison to *euo*prom, *hctA*prom, and *hctB*prom activities. Individual chlamydial cells with *euo* mRNA signal from five inclusions were identified using TrackMate, and the fluorescence intensity for each channel (mRNA and promoter reporter) was plotted (magenta dots). Individual chlamydial cells positive for *euo*prom, *hctA*prom, or *hctB*prom signal from five inclusions were also identified using TrackMate, and their expression intensity for each channel was plotted (green dots). **(B)** Z-projection confocal micrographs showing *hctA* mRNA localization in comparison to *euo*prom, *hctA*prom, and *hctB*prom activities. Individual chlamydial cells with *hctA* mRNA signal from five inclusions were identified using TrackMate, and the fluorescence intensity for each channel (mRNA and promoter reporter) was plotted (magenta dots). Individual chlamydial cells positive for *euo*prom, *hctA*prom, or *hctB*prom signal from five inclusions were also identified using TrackMate, and their expression intensity for each channel was plotted (green dots). **(C)** Z-projection confocal micrographs showing *hctB* mRNA localization in comparison to *euo*prom, *hctA*prom, and *hctB*prom activities. Individual chlamydial cells with *hctB* mRNA signal were identified from five inclusions using TrackMate, and the fluorescence intensity for each channel (mRNA and promoter reporter) was plotted (magenta dots). Individual chlamydial cells positive for *euo*prom, *hctA*prom, or *hctB*prom signal from five inclusions were also identified using TrackMate, and their expression intensity for each channel was plotted (green dots). The double-positive population was selected (box), and the percentage of the total for the mRNA+ cells (magenta) is indicated in each plot. Scale bar = 5 µm. RB, reticulate body; IB, intermediate body; EB, elementary body; FISH, fluorescence *in situ* hybridization.

To verify the association of the expression-grouped genes with specific cell forms, we used FISH to visualize gene expression in cells expressing GFP and RFP from developmental stage-specific promoters. To this end, we constructed two dual promoter-reporter constructs to delineate gene expression from the *euo*, *hctA*, and *hctB* promoters, which we have shown to be associated with RB, IB, and EB cell forms, respectively (Chiarelli et al., 2020; Chiarelli et al., 2023). We generated the strains L2-*hctB*prom-mScarlet_*euo*prom-neongreen (L2-BsciEng) and L2-*hctA*prom-mScarlet_*euo*prom-neongreen (L2-AsciEng), which express the RFP mScarlet-I from either the *hctB* promoter or *hctA* promoter along with the GFP protein Neongreen driven by the *euo* promoter. To validate our system, we visualized the mRNA expression of *euo*, *hctA*, and *hctB* in each strain using custom FISH probes (**Figure 4**). We infected the cells with L2-AsciEng and L2-BsciEng and processed them for each FISH probe at 24 hpi. Although we used dual promoter strains in these experiments, the data are presented in a single promoter format to simplify the presentation. We processed *Euo* and *hctA* data from L2-AsciEng samples, and we processed *hctB* data from L2-BsciEng samples. As expected, we observed *euo* mRNA primarily in the *euo*prom+ (RB) cells and not in either of the *hctA*prom+ (IB) or *hctB*prom+ (EB) cells (**Figure 4A**). We observed the *hctA* mRNA in a subset of cells that had overlap with the *hctA*prom signal but not the *euo*prom or *hctB*prom signal (**Figure 4B**). We observed the *hctB* mRNA in a subset of cells with overlap with the *hctA*prom+ and *hctB*prom+ cells but not *euo*prom+ cells (**Figure 4C**).

The TrackMate plugin in Fiji (Tinevez et al., 2014) was used to identify and quantify both the mRNA signal and promoter-reporter signal for each chlamydial cell in five inclusions from each infection. Cells were identified by their promoter-reporter signal (green) and also separately by their mRNA fluorescence signal (magenta). The fluorescence intensity was measured and plotted for both channels (FISH and promoter reporter) in both identified populations. Therefore, each plot represents two identified cell populations per reporter, the mRNA+ (magenta) population and their corresponding promoter-reporter fluorescence intensity, and the promoter reporter+ (green) population and their corresponding FISH signal. This analysis was performed for all three promoter reporters (*euo*p, *hctA*p, and *hctB*p) for each FISH mRNA probe (*euo*, *hctA*, and *hctB*) (**Figure 4**). The percentage of cells that were single or double positive for each signal was determined and presented in **Table 1**.

**Table 1:** Qualification of FISH and promoter signals for RB, IB, and EB genes.

Euo mRNA	Double positive	Single positive
euo mRNA/hctAp	7%	93%
hctAp/euo mRNA	7%	93%
euo mRNA/hctBp	1%	99%
hctBp/euo mRNA	5%	95%
euo mRNA/euop	90.0%	10.0%
euop/euo mRNA	91%	9%
**HctB mRNA**		
hctB mRNA/hctAp	59%	41%
hctAp/hctB mRNA	52%	48%
hctB mRNA/hctBp	28%	72%
hctBp/hctB mRNA	61%	39%
hctB mRNA/euop	20.0%	80.0%
euop/hctB mRNA	18%	82%
**HctA mRNA**		
hctA mRNA/hctAp	74%	26%
hctAp/hctA mRNA	77%	23%
hctA mRNA/hctBp	1%	99%
hctBp/hctA mRNA	32%	68%
hctA mRNA/euop	50.0%	50.0%
euop/hctA mRNA	28%	72%
**PorB mRNA**		
porB mRNA/hctAp	56%	44%
hctAp/porB mRNA	78%	22%
porB mRNA/hctBp	3%	97%
hctBp/porB mRNA	20.0%	80.0%
porB mRNA/euop	70.0%	30.0%
euop/porB mRNA	73%	27%
**SctJ mRNA**		
sctJo mRNA/hctAp	48%	52%
hctAp/sctJo mRNA	65%	35%
sctJo mRNA/hctBp	5%	95%
hctBp/sctJo mRNA	57%	43%
sctJo mRNA/euop	67%	33%
euop/sctJo mRNA	56%	44%

FISH, fluorescence *in situ* hybridization; RB, reticulate body; IB, intermediate body; EB, elementary body. Blue shading indicates IB, pink shading EB and green shading RB cell forms.

##### RB: *euo* FISH

We identified individual chlamydial cells expressing the *euo* mRNA in host cells that were infected with the promoter-reporter strains expressing fluorescent proteins from *euo*prom, *hctA*prom, and *hctB*prom. The analysis indicates that the *euo* mRNA+ cell population (magenta) when plotted for *euo*prom fluorescence and *euo* mRNA fluorescence was primarily double positive (90%) with high levels of both the *euo* FISH signal and *euoprom* signal. These *euo* mRNA+ cells from *hctAprom* infections were primarily single positive (93% single+ and 7% double+). This was also observed for the *hctB*prom infections; the *euo* mRNA+ cells when plotted for *euo* mRNA FISH signal intensity against *hctB*prom signal intensity were primarily single positive (99% single+ and 1% double+) (**Figure 4A**; **Table 1**).

We also used TrackMate to identify the promoter reporter-positive cell populations (green) and plotted the expression intensities of the promoter-reporter signals against the *euo* mRNA FISH signal. The *euo*prom+ cells were predominantly double positive (91%) (high *euo* mRNA signal, high *euo*prom signal), while the *hctA*prom+ cells and *hctB*prom+ cell populations (green) were predominantly single positive (7% and 5% double+, respectively) (low *euo* mRNA signal high *hctA*prom or *hctB*prom signal) (**Figure 4A**; **Table 1**).

##### IB: *hctA* FISH

The *hctA* mRNA-positive population was identified in the *euo*prom-, *hctA*prom-, and *hctB*prom-infected cells using TrackMate (magenta), and the intensities of the *hctA* mRNA FISH signal were plotted against each of the promoter-reporter intensity signals. The *hctA* mRNA+ cells from the *euo*prom infection were 50% single positive, likely due to carryover Neongreen protein from RB *euo*prom expression (**Figure 4B**; **Table 1**). The *hctA* mRNA+ cell population (magenta) was mostly double positive (74%) when compared to the *hctA*prom signal. Additionally, the *hctA* mRNA+ population had little to no *hctB*prom signal (1% double positive) (**Figure 4B**; **Table 1**).

We identified the promoter reporter-positive chlamydial cells (green) and plotted both the promoter-reporter signals and the FISH signal. The *euo*prom+ cell population (green) was mostly single positive (72%) with a small population of double-positive cells. The double-positive phenotype was presumably associated with carryover for the long-lived Neongreen protein (**Figure 4B**; **Table 1**). The *hctA*prom+ cell population (green) demonstrated a large double-positive sub-population (77%) as well as a single-positive sub-population that again was likely due to the long half-life of the mScarlet-I protein carried over into the EB population (**Figure 4B**; **Table 1**). The *hctB*prom+ cell population was mostly single positive with little *hctA* mRNA signal (68%) (**Figure 4B**; **Table 1**).

##### EB: *hctB* FISH

We performed the same analysis for the *hctB* mRNA+ cells (magenta). The *hctB* mRNA+ chlamydial cells were mostly single positive (80%) when compared to the *euo*prom signal with some detected long-lived Neongreen signal (**Figure 4C**; **Table 1**). The *hctB* mRNA+ cells were generally double positive for the *hctA*prom signal (59%) and a mix of single positive (72%) and double positive (28%) for the *hctB*prom signal (**Figure 4C**; **Table 1**).

For the promoter-reporter cells (green), the *euo*prom+ cells had a significant single-positive population (82%) and a smaller double-positive sub-population; in contrast, the *hctA*prom+ cells were both single and double positive for the *hctB* mRNA signal (52% double+ and 48% single+). In the *hctB*prom+ cells, there were also both single- and double-positive populations (61% double+ and 39% single+) (**Figure 4C**; **Table 1**).

The apparent disconnect between mRNA expression profiles and cognate fluorescent protein fluorescence for the *euo*, *hctA*, and *hctB* FISH results is not unexpected, as the fluorescent proteins have a much longer half-life than mRNA. Additionally, fluorescence from mScarlet-I and Neongreen proteins lags mRNA expression, as the proteins must be translated and then folded into the mature fluorescent state. Overall, these data indicate that, as expected, *euo* mRNA is expressed in RBs (double positive for the *euo*prom signal and *euo* mRNA signal), *hctA* mRNA is expressed in IBs (*hctA*prom+, *hctA* mRNA+, and *hctB*prom negative), and *hctB* mRNA is expressed in late IB/EBs (*hctB*prom+ and *hctB* mRNA+). Therefore, we used this workflow to interrogate cell form gene expression predicted by the RNA-seq clustering, binning, and volcano plot analysis.

#### Validation of *porB* as an IB gene

**Figure 5:**
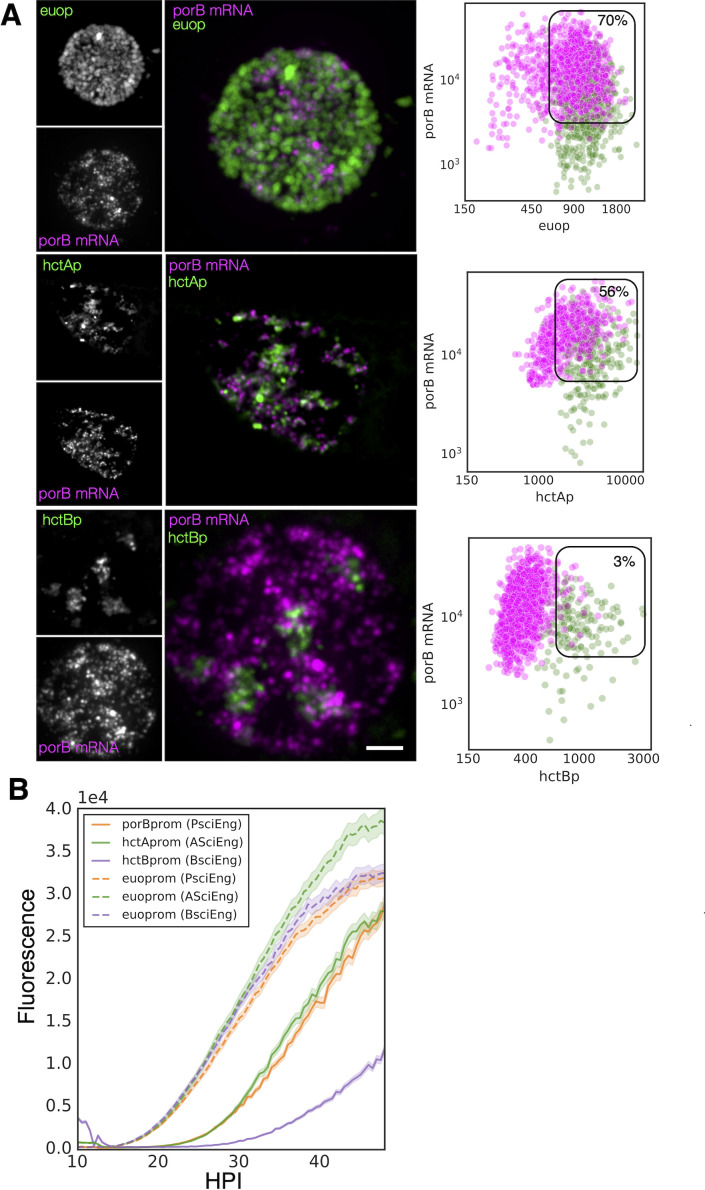
PorB gene expression is consistent with being classified as an IB gene. **(A)** FISH analysis of *porB* mRNA expression in comparison to *euo*prom, *hctA*prom, and *hctB*prom activities at 24 hpi. Confocal micrographs of L2-AsciEng and L2-BsciEng infected cells probed for *porB* mRNA expression from five inclusions using Molecular Instruments FISH probes. TrackMate was used to identify the *porB* mRNA+ cell population and measure the FISH fluorescent signal as well as the *euo*prom, *hctA*prom, and *hctB*prom fluorescent signals. The intensity for both channels for each cell was plotted (magenta dots). The *euo*prom, *hctA*prom, and *hctB*prom+ cell populations were also identified using TrackMate, and the signals from the FISH channel and fluorescent protein channels were plotted on the same graphs (green dots). The double-positive population was selected (box), and the percentage of the total for the mRNA+ cells (magenta) is indicated in each plot. **(B)** Cells were infected with L2-PsciEng, and the developmental gene expression kinetics were compared to those of L2-AsciEng and L2-BsciEng. The *euo*prom expression kinetics were comparable for all strains with expression first detected at ~15 hpi. *PorB*prom expression kinetics were nearly identical to *hctA*prom expression kinetics first detected at ~20 hpi, while *hctB*prom expression was initiated at ~26 hpi. Error cloud for fluorescent reporters represents SEM. n > 20 inclusions per strain. IB, intermediate body; FISH, fluorescence *in situ* hybridization.

Our previous studies showed that the *tarp*, *scc2*, and *hctB* promoters were all active much later than the *hctA* promoter (Chiarelli et al., 2020). The RNA-seq experiment presented here corroborates these data by placing the corresponding genes in the infectious EB category (**Figure 2C**). We also showed that the *hctA* promoter was active in a cell population distinct from the *hctB* promoter, making it a likely IB-expressed gene (Chiarelli et al., 2023). Here, we sought to verify an additional gene predicted to be expressed in IBs by the RNA-seq clustering experiment. We selected the porin gene *porB* (Kubo and Stephens, 2000), which clustered with the IB gene group as well as with a proven IB gene *hctA* (**Figure 2B**), for this analysis. We infected the cells with either L2-AsciEng or L2-BsciEng, fixed them at 24 hpi, and probed them for the *porB* mRNA. We took confocal images and viewed them as z-projections (**Figure 5A**). The *porB* mRNA signal (magenta) did not completely overlap with the *euo*prom+ signal (green) and appeared to be expressed in a subset of cells (**Figure 5A**). The *porB* mRNA had significant but not complete overlap with the *hctA*prom+ cells (green) and almost no overlap with the *hctB*prom+ cells (**Figure 5A**). Using the TrackMate protocol described above, we identified the promoter reporter-expressing populations, *euo*prom, *hctA*prom, and *hctB*prom (green) and then, separately, the *porB* mRNA+ population (magenta), and we measured the fluorescence intensity of each channel (fluorescent reporter proteins and mRNA signal within each population). The *porB* mRNA+ population (magenta) in the *euo*prom channel experiment was a mix of single- and double-positive cells (70% double+ and 30% single+) (**Figure 5A**; **Table 1**). This was also true for the *euo*prom+ population (green) (73% double+ and 27% single+). In comparison, the *porB* mRNA+ population (magenta) in the *hctA*prom channel experiment were also double positive and single positive (56% double+ and 44% single+). Additionally, the *hctA*prom+ population (green) was primarily double-positive cells (78%) (**Figure 5A**; **Table 1**). In the *hctB*prom channel experiment, the *porB* mRNA signal+ population (magenta) was primarily single positive (97%) and did not have appreciable *hctB*prom fluorescence. Conversely, the *hctB*prom+ population (green) was primarily single positive 80% with low *porB* mRNA signal. Taken together, the *porB* mRNA expression pattern was similar to that of the *hctA* mRNA expression pattern, strongly suggesting that *porB* is expressed primarily in the IB cell form.

Next, the kinetics of the activity of the *porB* promoter were evaluated to determine if the kinetics were similar to those of the *hctA* promoter (Chiarelli et al., 2020; Chiarelli et al., 2023). The *hctA* promoter of AsciEng was replaced with the promoter region of *porB* (−137 to +30 bp) and transformed into *Ctr* to create L2-PsciEng. Live cell imaging was used to measure the expression of Neongreen driven by *euo*prom and mScarlet-I driven by *porB*prom. Cells were infected with PsciEng at an multiplicity of infection (MOI) ~ 0.3 and imaged for both the Neongreen and mScarlet-I fluorescence at 10 hpi every 30 minutes for a further 48 hours. For comparisons, L2-AsciEng and L2-BsciEng strains were imaged in parallel, as we have previously shown that *euo* promoter activity is detected at ~15 hpi followed by the *hctA* promoter and finally the *hctB* promoter (Chiarelli et al., 2020; Chiarelli et al., 2023). The kinetics of the *porB* promoter mirrored those of the *hctA* promoter. *Euo*prom activity was detected at ~15 hpi followed by the activity of *porB*prom and *hctA*prom at ~20 hpi and by *hctB*prom activity starting at ~26 hpi (**Figure 5B**).

Cell form-specific promoter activity was also evaluated in the L2-PsciEng strain (**Supplementary Figure S2**). Cells were infected with L2-PsciEng, fixed at 16 and 24 hpi, and evaluated using confocal microscopy. At 16 hpi, the inclusion contained primarily *euo*prom+ cell forms (bright green) and little to no *porB*prom signal. The inclusions at 24 hpi contained both a *euo*prom+ subset of chlamydial cells as well as a subset of cells that were *porB*prom+ (**Supplementary Figure S2**). Taken together, these data suggest that *porB*, as predicted by the RNA-seq clustering data, can be considered an IB gene.

#### Predicted cell type expression of T3SS genes

**Figure 6:**
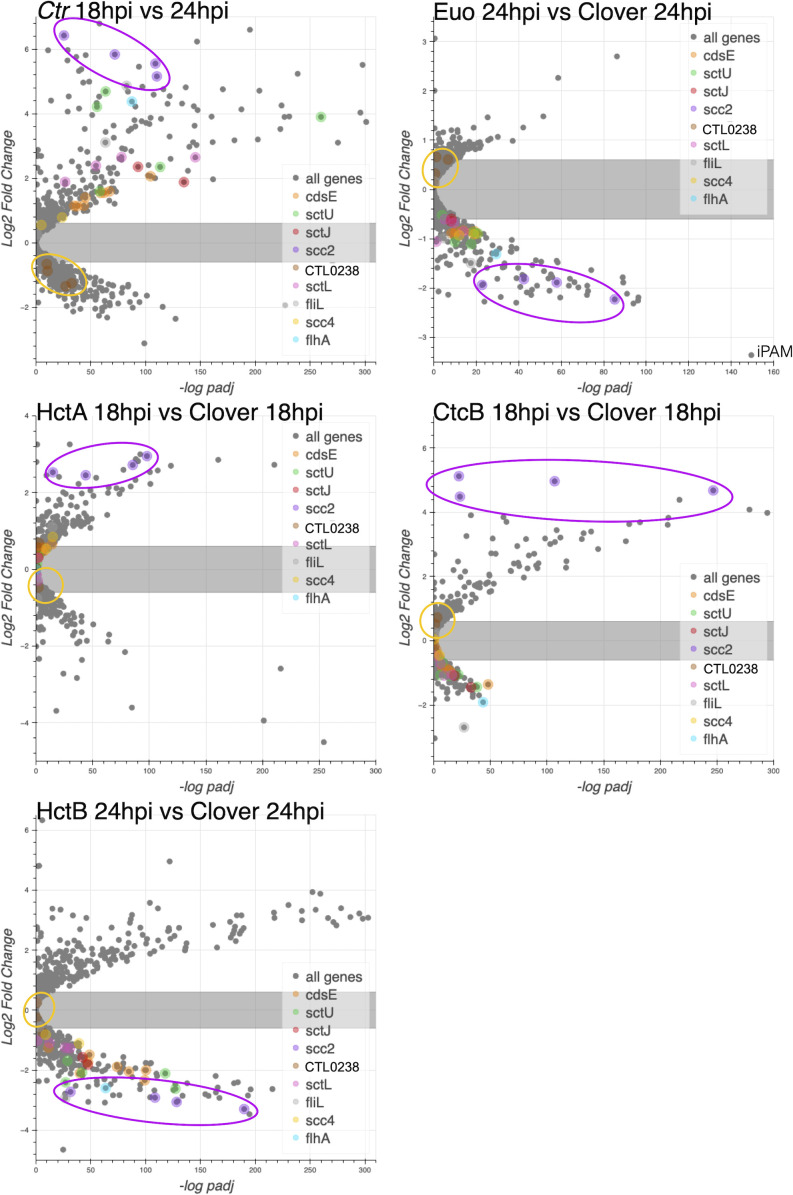
The effects of ectopic expression of Euo, HctA, CtcB, and HctB on T3SS structural genes. The log2fold change RNA-seq differential expression data from the ectopic expression experiments were plotted against the −log of the p-value (−log padj), and the operons for the T3SS were highlighted. For the wt *Ctr* 18 hpi vs. 24-hpi samples, most of the T3SS structural genes were upregulated, while for the Euo ectopic expression experiment, most of these operons were downregulated. Again, like the IB genes in the HctA and CtcB ectopic expression experiments, most of the structural genes were downregulated or unchanged. Two operons did not follow this pattern: *CTL0238*-op and *scc2*-op. Both operons encode the components of the T3SS translocon. The four genes in the *CTL023*8-op (gold circle) were regulated like RB genes, while the four genes in *scc2*-op (purple circle) were regulated like EB genes. T3SS, type III secretion system; IB, intermediate body; EB, elementary body; RB, reticulate body.

We noticed an intriguing expression pattern of the T3SS structural genes in the gene expression profile data that suggested cell form-specific expression (**Figures 2B, C**). All chlamydial genomes contain genes encoding the ubiquitously conserved core components of the apparatus as reviewed by Ferrell et al (Ferrell and Fields, 2016). To explore cell type specificity, we plotted the effects of the ectopic expression of each of the four regulatory proteins on the T3SS operons. We used our wt RNA-seq data (Grieshaber et al., 2018), operon prediction software (Price et al., 2005), and the RT-PCR data published by Hefty et al (Hefty and Stephens, 2007) to annotate the T3SS operons (**Supplementary Table S2**) and plotted the expression data using volcano plots. These plots revealed that the majority of the T3SS operons were regulated in an IB-like pattern of gene expression, i.e., upregulated between 18 and 24 hpi, repressed by Euo and HctB ectopic expression, but not induced by the CtcB or HctA ectopic expression (**Figure 6**). Interestingly, the exception to this pattern was the two operons for the T3SS translocons: [*CTL0238*, *lcrH*, *copB_2*, and *copD_2* (*CTL0238*-op)] and [*scc2*, *CTL0840*, *copB*, and *copD* (*scc2*-op)] (**Supplementary Table S2**). The chlamydial genome contains two separate operons for the translocon (Ferrell and Fields, 2016; Stephens et al., 1998). The four genes in *CTL0238*-op were regulated like RB genes, while the four genes in *scc2*-op were regulated like EB genes (**Figure 6**).

#### Validation of cell type expression of T3SS structural operons by FISH

**Figure 7:**
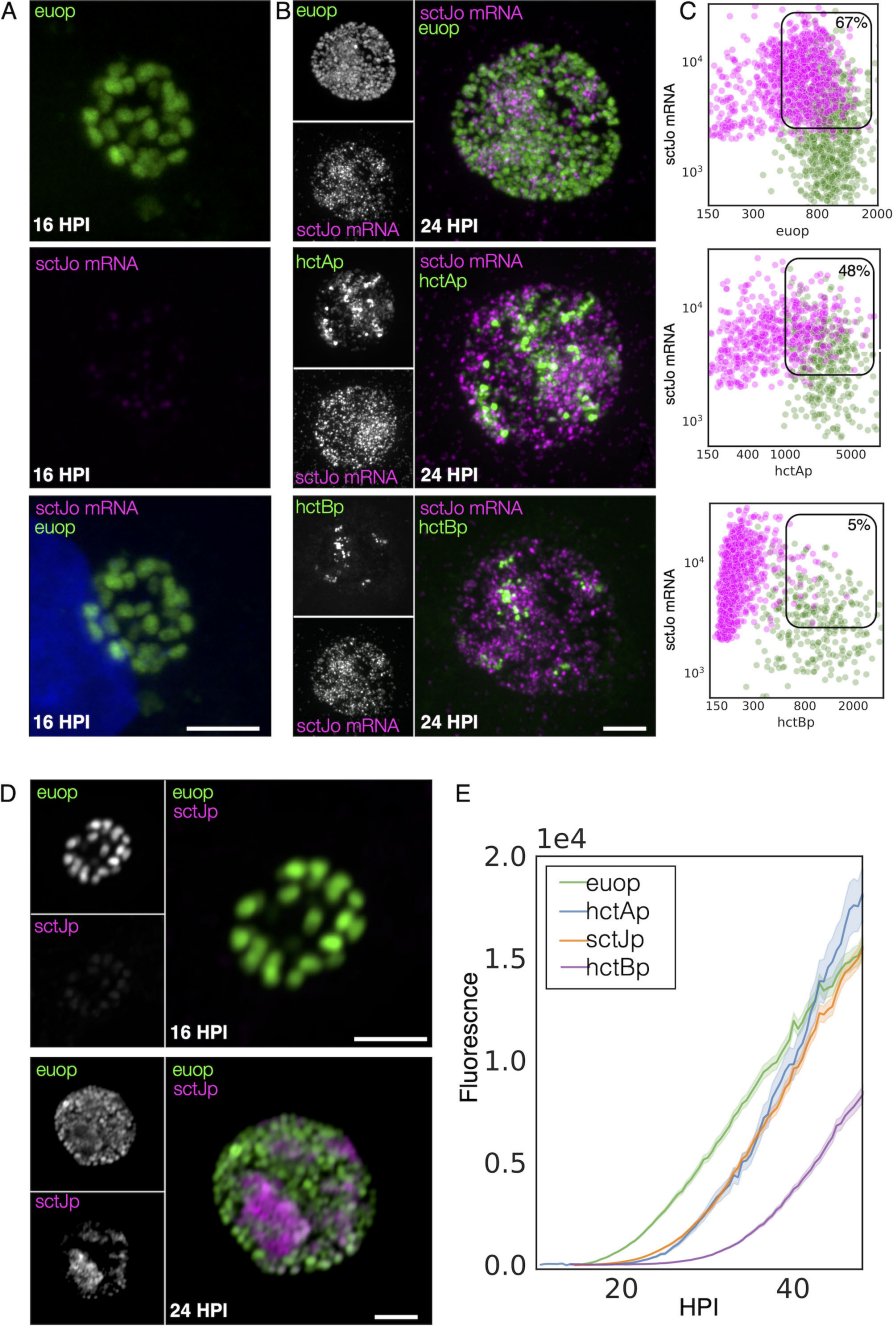
IB cell type expression of the T3SS structural operon *SctJ-*op. **(A)** Cells were infected with L2-AsciEng for 16 hpi, fixed, and stained using a FISH probe (*sctL* to *sctR*) to the mRNA for the T3SS structural operon *sctJ*-op, the RB control *euo*, and the IB control *hctA*. All cells were positive for *euo*prom expression (green). The FISH-stained cells were only positive for *euo* mRNA (magenta) and were negative for *hctA* mRNA (magenta) and *sctJ-op* mRNA (magenta). **(B)** Cells were infected with L2-AsciEng and L2-BsciEng for 24 hpi, fixed, and stained using FISH for the *sctJ*-op mRNA. For the *euo*prom sample, the *sctJ*-op FISH signal (magenta) was present in a distinct subset of cells and not in the majority of the *euo*prom+ cells (green). **(C)** TrackMate was used to identify the *sctJ*-op mRNA+ cells from five inclusions, and the signals for *euo*prom and FISH were quantified for each *sctJ*-op+ cell and plotted (magenta dots). The converse was also performed, the *euo*prom+ cells were identified, and the *euo*prom signal and FISH signal were quantified for each *euo*prom+ cell and plotted (green dots). The FISH signal was also compared to the *hctA*prom expression pattern and showed subsets of cells that were stained for both *sctJ*-op mRNA and *hctA*prom expression as well as non-overlapping populations. The *sctJ*-op mRNA+ cells were again identified using TrackMate, and the signals for *hctA*prom and FISH were quantified for each *sctJ*-op+ cell and plotted (magenta dots). Each *hctA*prom+ cell was also identified, and the FISH and *hctA*prom signals were determined and plotted (green dots). The *sctJ-*op FISH staining was also compared to the expression from the *hctB*prom reporter. The *sctJ*-op mRNA FISH staining was again present in a subset of cells but showed little overlap with the *hctB*prom fluorescent signal. The FISH signal and *hctB*prom signal were measured in both cell populations (*sctJo* mRNA+ cells and *hctB*prom+ cells) and plotted; *sctJo* mRNA+ cells in magenta dots and *hctB*prom+ cells in green dots. Both populations were primarily single positive, either *sctJ*-op mRNA high or *hctB*rpom high, but rarely both. The double-positive population for mRNA+ cells was selected (box), and the percentage of the total is indicated. **(D)** Cos-7 cells infected with L2-JsciEng (*sctJ* promoter driving scarlet-I) were fixed at 16 and 24 hpi and imaged. The 16-hpi inclusions contain primarily *euo*prom-expressing cells (green) with little *sctJ*prom scarlet-I signal. At 24 hpi, there are two dominant cell populations, *euo*prom+ and *sctJ*prom+ cells. Scale bar = 5 µm. **(E)** The kinetics of *sctJ*prom activity were determined and compared to those of *euo*prom, *hctA*prom, and *hctB*prom. Cos-7 cells infected with L2-JsciEng, L2-AsciEng, and L2-BsciEng and imaged every 30 minutes starting at 10 until 48 hpi. The *euo*prom signal began to increase at ~15 hpi, while the *sctJ*prom and *hctA*prom signals began to increase at ~22 hpi followed by the *hctB*prom activity at 28 hpi. IB, intermediate body; T3SS, type III secretion system; FISH, fluorescence *in situ* hybridization; RB, reticulate body.

Next, the cell type expression of two of the T3SS operons predicted to be expressed in the IB, the *sctU* operon, and the *sctJ* operon was investigated (**Supplementary Table S2**). The *sctU* operon (*sctU*-op) encodes the genes *sctU*, *sctV*, *lcrD*, *copN*, *scc1*, and *malQ*, while the *sctJ* operon (*sctJ*-op) includes the genes *sctJ*, *sctK*, *sctL*, *sctR*, *sctS*, and *sctT*. Custom FISH probes were used for *sctU* through *lcrD* for the detection of the *sctU-op* mRNA and *sctL* to *sctR* for the detection of *sctJ*-op mRNA. Cell monolayers were infected with L2-AsciEng and L2-BsciEng at an MOI ~0.3 and processed for FISH staining at 16 and 24 hpi (**Figure 7**, sctJo; **Supplementary Figure S3**, *sct*Uo). The FISH signal was not observed in the RB cells (*euo*prom+) at 16 hpi for either *sctU*-op (**Supplementary Figure S3A**) or *sctJ*-op (**Figure 7A**). In the infections fixed at 24 hpi, the FISH staining for both operons was observed in cells distinct from the *euo*prom+ and *hct*Bprom+ cells (**Figure 7B**, sctJo; **Supplementary Figure S3B**, *sctU*o). However, both T3SS operon mRNAs were detected in a subset of the *hctA*prom+ cell population (**Figure 7B**, sctJo; **Supplementary Figure S3B**, *sctU*o). Again, our TrackMate workflow was used to quantitate these data. Cells were identified by their promoter-reporter signal (green) and separately by their mRNA/FISH signal (magenta). The fluorescence intensity was measured and plotted for both channels [FISH (magenta) and promoter reporter (green)] in both identified populations as described in **Figure 4**. As the *sctJ*-op and *sctU*-op results were very similar, only the sctJ-op data analysis is discussed in detail below.

##### *sctJ*-op: expression in RB cells

We identified the mRNA+ cell population (magenta) and quantified both the *euo*prom signal intensity and the FISH signal intensity. For the *sctJ*-op mRNA+ cells, there was both a double-positive population (high mRNA signal and high *euo*prom signal) and a single-positive population (67% double and 33% single+). We also quantified the mRNA expression in RBs by identifying the *euo*prom+ cells (green) and measuring the *sctJ*-op FISH signal and plotted this against the *euo*prom signal intensity (**Figure 7C** *euo*p, **Table 1**). The *euo*prom+ population (green) was both single and double positive for both operons (56% double+ and 44% single+) (**Figure 7C** *euo*p, **Table 1**). At 16 hpi, there was no measurable *sctJ* mRNA signal in any of the cells.

##### *sctJ*-op: expression in IB cells

The *sctJ*-op mRNA+ cell population (magenta) was identified, and both the *hctA*prom signal intensity and the FISH signal intensity were quantified and plotted. For the *sctJ*-op mRNA+ cells (magenta), there was both a double-positive population and a single-positive population (48% and 52%, respectively) (**Figure 7C** *hctA*p, **Table 1**). For the *hctA*prom+ cell population (green), there were both single- (*hctA*prom) and double-positive (*hctA*prom and mRNA) populations (65% and 35%, respectively) (**Figure 7C** *hctA*p, **Table 1**). These data further suggest that *sctU*-op and *sctJ*-op were expressed in the IB cell type. It is likely that the *hctA*prom+ single-positive population is late IB/EB cell forms that are becoming EBs and have repressed *sctJ*-op expression.

##### *sctJ*-op: expression in EB cells

In contrast, the mRNA+ cell populations for the *sctJ*-op in the *hctB*prom-expressing cells were distinct single-positive (mRNA signal) populations (5% double+ and 95% single+) (**Figure 7C** *hctB*p, **Table 1**). Additionally, the *hctB*prom+ cell population was also primarily single positive (*hctB*prom). These data suggest that *sctJ*-op was not expressed in the EB cell forms. Combined, these overall expression patterns of the *sctJ* operon were very similar to those of the *hctA* mRNA and *porB* mRNA FISH, supporting an IB-like gene expression pattern.

To determine cell type specificity for the expression of the *sctJ* operon, the *hctA* promoter in the AsciEng construct was replaced with the *sctJ* promoter (120 bp upstream of the ATG start of *sctJ*) and transformed into *Ctr* L2, creating L2-JsciEng. Cells were infected with L2-JsciEng and fixed at 16 and 24 hpi, and cell form specificity was evaluated using confocal microscopy. At 16 hpi, only the Neongreen signal was detected (**Figure 7D**). There were two obvious cell populations present at 24 hpi, one brightly expressing the Neongreen protein from the *euo* promoter and a second population that was brightly expressing the mScarlet-I protein from the *sctJ* promoter (**Figure 7D**). In addition to confocal microscopy, live cell imaging was used to measure the kinetics of the expression of Neongreen driven by the *euo* promoter and mScarlet-I driven by the *sctJ* promoter. Cells were infected with L2-JsciEng at an MOI ~0.3 and imaged for both Neongreen and mScarlet-I fluorescence every 30 minutes from 10 hpi until 48 hpi. For comparisons, L2-AsciEng and L2-BsciEng strains were imaged in parallel (Chiarelli et al., 2020; Chiarelli et al., 2023). The kinetics of the *sctJ*prom activity mirrored those of *hctA*prom (**Figure 7E**). Overall, these data support the observation that the *sctJ* and *sctU* operons are expressed primarily in the IB cell form.

#### FISH-based analysis of cell type expression of the T3SS translocon operons

**Figure 8:**
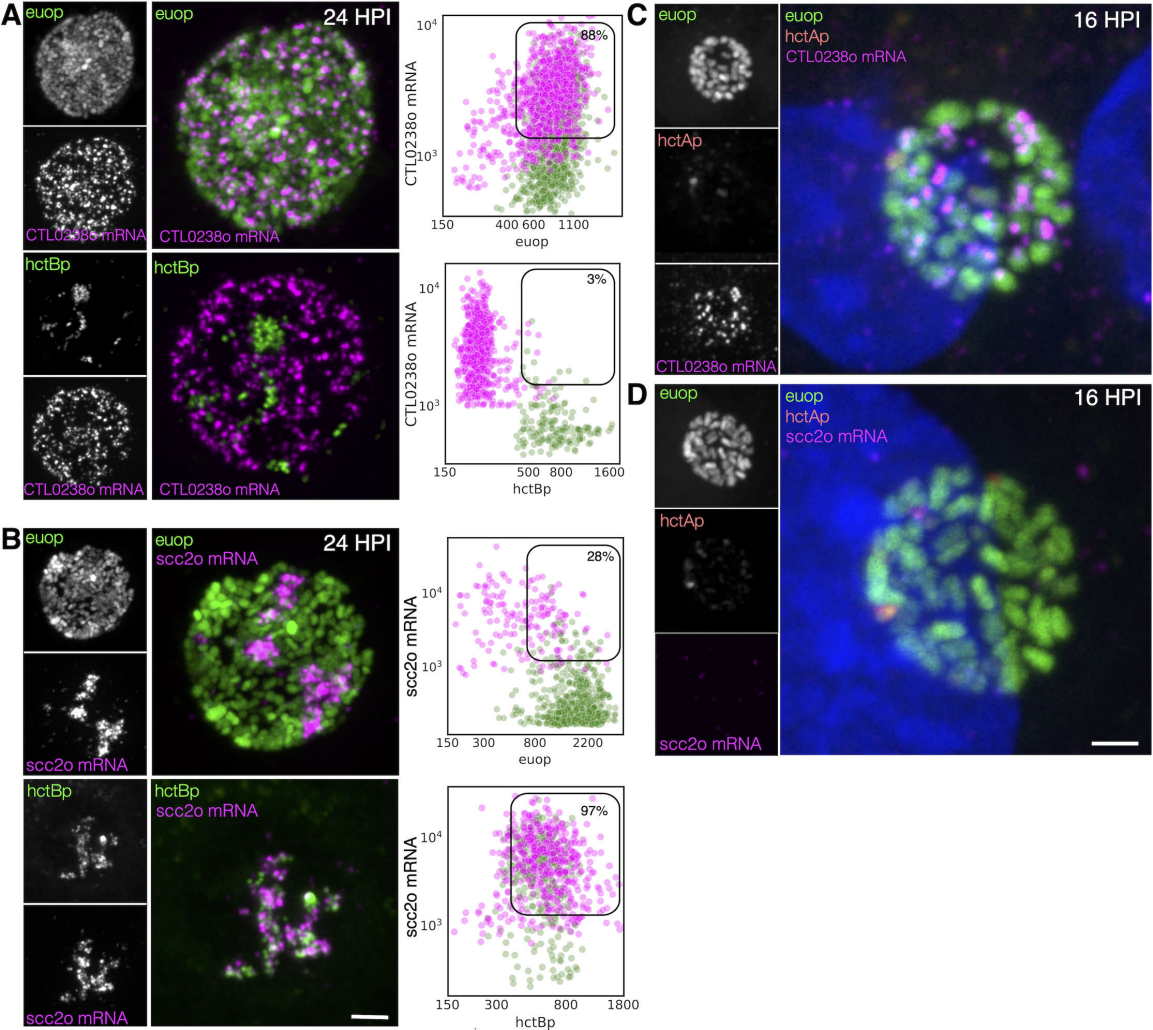
Cell type expression of the two T3SS translocons. **(A)** Cell monolayers were infected with L2-BsciEng for 24 hpi and stained for the mRNA expression of the *CTL0238*-op using FISH (magenta), *euo*prom expression (green), and *hctB*prom expression (green). Individual chlamydial cells with *CTL0238*-op mRNA signal from five separate inclusions were identified using TrackMate, and the fluorescence intensity for each channel (mRNA and promoter reporter) was plotted (magenta dots). Individual chlamydial cells positive for *euo*prom or *hctB*prom signal from five separate inclusions were also identified using TrackMate, and the expression intensity for each channel (mRNA and promoter reporter) was plotted (green dots). **(B)** Cos-7 cells were infected with L2-BsciEng for 24 hpi and stained for the mRNA expression of *scc2*-op using FISH. *Scc2*-op FISH signal in magenta; *euo*prom and *hctB*prom signals in green. Individual chlamydial cells positive for *scc2*-op mRNA signal from five inclusions were identified using TrackMate, and the fluorescence intensity for each channel (mRNA and promoter reporter) was plotted (magenta dots). Individual chlamydial cells positive for *euo*prom or *hctB*prom signal from five inclusions were also identified using TrackMate, and the expression intensity for each channel (mRNA and promoter reporter) was plotted (green dots). The double-positive population for mRNA+ cells was selected (box), and the percentage of the total is indicated. **(C)** Host cells infected with AsciEng and fixed at 16 hpi were probed for *CTL0238*-op mRNA and *scc2*-op mRNA **(D)** expression using FISH. *Euo*prom expression (green) had significant overlap with *CTL0238-*op mRNA signal. For *scc2*-op, the FISH signal was undetected. Scale bar = 5 µm. T3SS, type III secretion system; FISH, fluorescence *in situ* hybridization.

As mentioned above, the *Ctr* genome encodes two operons for the T3SS translocon each of which contains four genes: *CTL0238*-op and *scc2*-op (Stephens et al., 1998). This duplication is conserved in all the vertebrate-infecting chlamydial species. The expression profiles from our clustering data and volcano plots suggested that the two translocon operons are expressed in different cell types: the *CTL0238*-op in RBs and the *scc2*-op in EBs (**Figure 6**). To verify differential cell type expression, host cells were infected with L2-BsciEng, fixed at 24 hpi, and probed with custom FISH probes designed against C*TL0238*-op and *scc2*-op. Confocal micrographs showed that the mRNA FISH signal for *CTL0238*-op heavily overlapped with the *euo*prom channel but was distinct from the *hctB*prom+ cells (**Figure 8A**, *CTL0238*-op mRNA). In contrast, the mRNA signal for *scc2*-op was distinct from the *euo*prom+ cells but almost completely overlapped the *hctB*prom+ cells (**Figure 8B**, *scc2*-op mRNA).

This expression pattern was again quantified using our TrackMate workflow. Chlamydial cells were identified by their promoter-reporter signal (green) and then separately by their mRNA fluorescence signal (magenta). The fluorescence intensity was measured and plotted for both channels: FISH (magenta) and promoter reporter (green).

#### *CTL0238*-op: expression in RB cells

We identified the *CTL0238*-op mRNA+ cell population and quantified both the *euo*prom signal intensity and the FISH signal intensity. For the *CTL0238*-op mRNA+ cells, there was primarily a double-positive population (88%) (high mRNA signal and high *euo*prom signal) (**Figure 8A**; **Table 2**). We also quantified the mRNA expression in RB cells (*euo*prom+ cells). We plotted the *CTL0238*-op FISH signal against the *euo*prom signal intensity, and the *euo*prom+ cells were mostly double positive (71%) with an additional single-positive population (29%) (**Figure 8A**; **Table 2**). This single-positive (*euo*prom+, *CTL0238*-op mRNA−) population is likely due to the long half-life of the GFP protein.

**Table 2:** Quantification of FISH and promoter signals for the type III secretion system.

CTL0238o mRNA	Double positive	Single positive
CTL0238o mRNA/hctBp	3%	97%
hctBp/CTL0238o mRNA	11%	89%
CTL0238o mRNA/euop	88%	12%
euop/CTL0238o mRNA	71%	29%
**Scc2o mRNA**		
Scc2o mRNA/hctBp	97%	3%
hctBp/Scc2o mRNA	89%	11%
Scc2o mRNA/euop	28%	72%
euop/Scc2o mRNA	10.0%	90.0%
**incD mRNA**		
incD mRNA/hctBp	2%	98%
hctBp/incD mRNA	25%	75%
incD mRNA/euop	94%	6%
euop/incD mRNA	90.0%	10.0%
**incV mRNA**		
incV mRNA/hctBp	98%	2%
hctBp/incV mRNA	60.0%	40.0%
incV mRNA/euop	24%	76%
euop/incV mRNA	3%	97%

FISH, fluorescence *in situ* hybridization. Blue shading indicates IB, pink shading EB and green shading RB cell forms.

##### *CTL0238*-op: expression in EB cells

In contrast, the *CTL0238*-op mRNA+ cell population when plotted for the mRNA signal and the *hctB*prom signal was a distinct single-positive population (97%) (*CTL0238*-op mRNA+, *hctB*prom−) (**Figure 8A**; **Table 2**). We also identified the *hctB*prom+ cell population and plotted the mRNA signal and *hctB*prom signal. This population was also primarily single positive (89% (*hctB*prom+, *CTL0238*-op mRNA−) (**Table 2**).

##### *scc2*-op

The *scc2-op* mRNA FISH quantification showed the opposite results (**Figure 8B**). The *scc2-op*+ mRNA cells were primarily single positive when plotted against the *euo*prom signal (28%) and double positive when plotted against the *hctB*prom signal (97%) (**Figure 8B**, **Table 2**). The *euo*prom+ cell population was only 10% double positive, while the *hctB*prom+ cells were primarily double positive (89%) for *scc2-op*+ mRNA (**Figure 8B**; **Table 2**).

To further highlight the differential expression of *CTL0238*-op mRNA and *scc2*-op mRNA, we infected cells with L2 AsciEng and processed the samples for FISH at 16 hpi when most of the chlamydial cells were RBs. As expected, at 16 hpi, essentially all the cells were green RBs (*euo*prom+) with little to no red IB (*hctA*prom+) cells. The *euo*prom+ cells were all positive for the *CTL0238*-op FISH signal (**Figure 8C**, *CTL0238*-op). In contrast, the *scc2*-op FISH signal was undetectable in the *euo*prom+ cells at 16 hpi (**Figure 8D**, *scc2*-op).

Taken together, these data support the observation that the two translocon operons are differentially regulated and are expressed in distinct cell forms. *scc2*-op is expressed in late IB/EB cells, while *CTL0238*-op is expressed in RB cells.

### Predicted cell type expression of T3SS effectors

**Figure 9:**
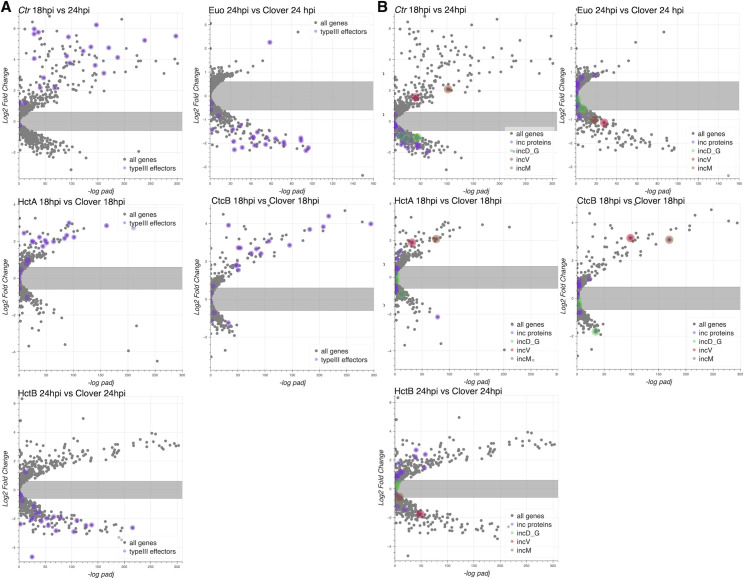
Effects of ectopic expression of Euo, HctA, CtcB, and HctB on the expression of T3SS effectors. RNA-seq differential expression data (log2fold change) plotted vs. the −log of the p-value (−log padJ) for *Ctr* ectopically expressing Euo, HctA, CtcB, and HctB. **(A)** The T3SS effectors are highlighted in purple. **(B)** All the *inc* protein genes are highlighted in purple, while the genes for the *incD*–*G* operon are highlighted in green; *incV* and *incM* are highlighted in red and orange, respectively. T3SS, type III secretion system.

In general, T3SS translocons are involved in interacting with host membranes to facilitate the secretion of T3SS effectors into target cells (Dey et al., 2019). During infection, *Ctr* secretes effectors into/through two membrane systems, the host cell plasma membrane, and, once inside the cell, the chlamydial inclusion membrane. Additionally, the chlamydial T3SS is known to secrete different kinds of effectors, soluble proteins, and integral membrane inclusion (Inc) proteins (Dehoux et al., 2011; Ferrell and Fields, 2016; Peters et al., 2005; Betts-Hampikian and Fields, 2010). Using volcano plots, we asked how the genes encoding the soluble effector proteins (**Supplementary Table S3**) and Inc proteins (**Supplementary Table S4**) were regulated by the ectopic expression of Euo, HctA, HctB, and CtcB. The vast majority of the soluble T3SS effector genes were regulated like EB genes: higher in 18–24 hpi and induced by HctA and CtcB ectopic expression but downregulated by Euo and HctB ectopic expression (**Figure 9A**). In contrast, most of the *incs* were expressed as RB genes except for *incM* and *incV*, which were expressed like EB genes (**Figure 9B**).

#### Cell type expression of *inc* genes by FISH

**Figure 10:**
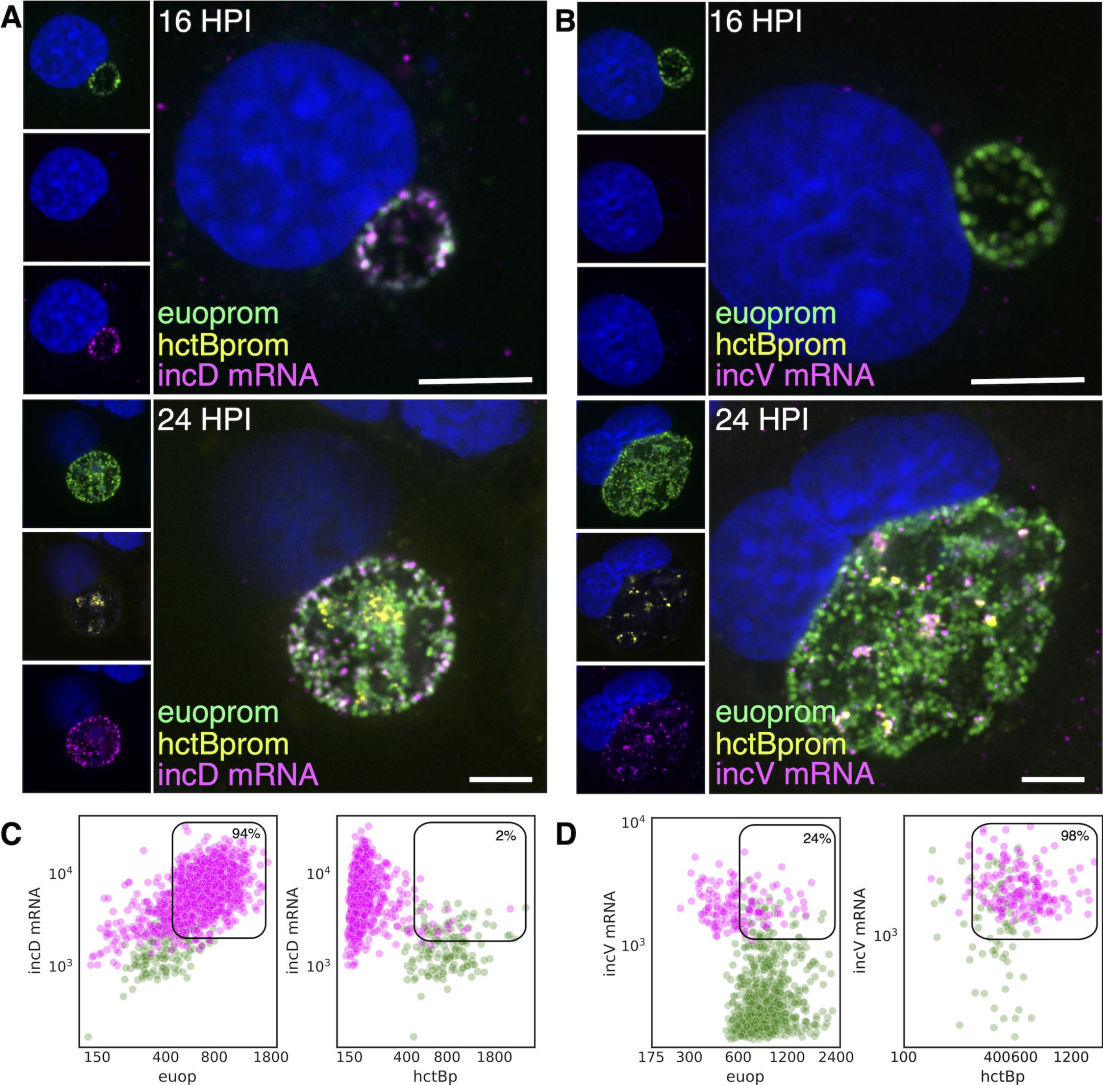
Cell type expression of *incD* and *incV*. Cos-7 cells infected with L2-BsciEng for 16 and 24 hpi and stained for *incD* and *incV* mRNA expression using custom FISH probes. **(A)** The *incD* mRNA (magenta) was visibly expressed in the *euo*prom+ (green) RB cells at 16 hpi, while *hctB*prom signal was not detected. At 24 hpi, the *incD* mRNA signal (magenta) overlapped with the *euo*prom signal (green) but was separate from the *hctB*prom+ cells (yellow). **(B)** The *incV* mRNA signal (magenta) was undetected at 16 hpi. At 24 hpi, the *incV* mRNA signal showed overlap with the *hctB*prom signal (yellow) but not the *euo*prom signal (green). **(C)** Individual chlamydial cells positive for *incD* mRNA signal were identified from five separate inclusions at 24 hpi using TrackMate, and the fluorescence intensity for each channel (mRNA and promoter reporter) was plotted (magenta dots). Individual chlamydial cells positive for *euo*prom or *hctB*prom signal were also identified using TrackMate, and the expression intensity for each channel (mRNA and promoter reporter) was plotted (green dots). **(D)** Individual chlamydial cells positive for *incV* mRNA signal from five separate inclusions at 24 hpi were identified using TrackMate, and the fluorescence intensity for each channel (mRNA and promoter reporter) was plotted (magenta dots). Individual chlamydial cells positive for *euo*prom or *hctB*prom signal were also identified using TrackMate, and the expression intensity for each channel (mRNA and promoter reporter) was plotted (green dots). The double-positive population for the mRNA+ cells was selected (box), and the percentage of the total is indicated. Scale bar = 5 µm. FISH, fluorescence *in situ* hybridization.

The volcano plots suggested that the majority of the *inc* effector genes were expressed in RBs. However, two *inc* genes (*incV* and *incM*) stood out as potential EB genes (**Figure 9B**). To determine if the putative late Incs, *incV* and *incM*, were expressed late in RBs or were bona fide EB genes, we compared mRNA expression of a known RB expressed Inc, *incD*, to the expression of *incV* and *incM* using FISH. Cells infected with L2-BsciEng were probed for the expression of *incD*, *incV*, and *incM* mRNA at 16 hpi (mostly RBs) and 24 hpi (all three cell forms). Confocal microscopy revealed that, as expected, *incD* was expressed in *euo*prom+ RB cells at both 16 and 24 hpi and not in *hctB*prom+ EBs present at 24 hpi (**Figure 10A**). Conversely, *incV* and *incM* mRNAs could not be detected at 16 hpi (no EBs) and were expressed exclusively in *hctB*prom+ EBs (**Figure 10B**, *incV* and **Supplementary Figure S4**, *incM*).

Next, the expression of *incD* and *incV* in RBs and EBs from inclusions from the 24-hpi experiments was quantified using our TrackMate workflow. Chlamydial cells were identified by their promoter-reporter signal (green) and separately by their mRNA fluorescence signal (magenta), and the signal for both populations was plotted.

##### *incD*: expression in RB cells

We identified the *incD* mRNA+ cell population (magenta) and plotted both the *euo*prom signal intensity and the FISH signal intensity (**Figure 10C**; **Table 2**). For the incD mRNA+ cells, there was primarily a double-positive population (94%) (high *incD* mRNA signal and high euoprom signal). We also quantified the mRNA expression in RB cells (euoprom+ cells). We plotted the *incD* FISH signal against the *euo*prom signal intensity, and the *euo*prom+ cells were mostly double positive (90%).

##### *incD*: expression in EB cells

In contrast, the *incD* mRNA+ cell population when plotted for the mRNA signal and the *hctB*prom signal was a distinct single-positive population (98%) (*incD* mRNA+, *hctB*prom−) (**Figure 10C**; **Table 2**). We also identified the *hctB*prom+ cell population and plotted the mRNA signal and *hctB*prom signal. This population was primarily single positive (75%) (*hctB*prom+, *incD* mRNA−).

##### incV

We only analyzed and plotted the *incV* data, as *incV* and *incM* showed similar FISH results. The *incV*+ cells were primarily single positive (76%) when plotted against the *euo*prom signal and double positive when plotted against the *hctB*prom signal (98%) (**Figure 10D**; **Table 2**). The *euo*prom+ cell population was also primarily single positive (97%), and the *hct*Bprom+ cells were primarily double positive (60%) (**Figure 10D**; **Table 2**). These data support the hypothesis that *incD* is indeed an RB gene and that *incV* and *incM* are EB genes.

## Discussion

The chlamydial developmental cycle has traditionally been defined by the timeline of the infection. The infectious EB invades the host cell and differentiates into the RB cell form, which then begins to divide. The genes involved in this process have been described as the early genes. After EB-to-RB differentiation, RBs replicate, and the gene expression associated with this timeframe is usually considered the chlamydial mid-cycle. Genes that are upregulated from ~24 hpi until cell lysis, when EBs accumulate in the inclusion, are considered late genes (Shaw et al., 2000; Nicholson et al., 2001; Belland et al., 2003). We have dissected the developmental cycle and developed a model based on cell type transitions (Chiarelli et al., 2023). Our model suggests that the developmental cycle is best described by a programmed cell production model (Chiarelli et al., 2023). In this model, the EB enters the host cell (through the use of premade effectors) and initiates immediate early protein synthesis (EB-to-RB differentiation genes) to begin the EB-to-RB differentiation process. The EB-to-RB differentiation process takes ~10 hours to complete. The completion of EB-to-RB differentiation is defined by the first division of the nascent cell resulting in RB cells. At this stage, the RBs expand in number through cell division, amplifying the infection. Our model suggests the RBs mature during this amplification stage, ultimately producing daughter cells with asymmetric fates. One daughter cell becomes the IB cell form, while the other remains an RB. Our model defines the IB as the cell type committed to EB formation. The mature RBs at this stage continue to replicate, producing one IB and one RB. The IBs never re-enter the cell cycle and instead transition into the infectious EB, which takes ~10 hours to complete (Chiarelli et al., 2023).

In this study, we ectopically expressed four transcriptional regulatory proteins that all blocked the progression of the developmental cycle. We determined the effects of the expression of these regulatory proteins using RNA-seq and compared them using a clustering algorithm, which resulted in three distinct regulation patterns. The first cluster contained genes that were unaffected by the ectopic expression of Euo and were not upregulated between 18 and 24 hpi of a *Ctr* L2 wt infection. The second cluster consisted of genes whose expression increased from 18 to 24 hpi of a wt infection but were not induced by the ectopic expression of HctA or CtcB. The third cluster of genes was upregulated between 18 and 24 hpi and by the ectopic expression of both HctA and CtcB. These groups fit well into the major cell categories in our model: RBs, IBs, and EBs. Using the clustering observation, we created selection criteria based on changes in gene expression from our RNA-seq experiments. We were able to categorize 639 of 902 genes (70%) into one of the RB, EB, or IB categories. The genes that we could not assign were either expressed at levels too low to have confidence in the expression pattern or had a unique expression pattern that did not fit into the three categories, suggesting potential unique roles in chlamydial biology. This study focused on determining gene expression by measuring mRNA, and it remains to be determined if any of these genes are translationally regulated as well.

The RB cell is the replicating cell form leading to the expansion of cell numbers. Based on the changes in gene expression after the ectopic expression of Euo, HctA, CtcB, or HctB, we found that 532 genes were regulated as RB genes. This category included cell replication genes, genes involved in protein synthesis, genes for many of the Inc proteins, and *euo*. Based on our selection criteria, this group likely encompasses both potential constitutive genes (expressed in RBs, IBs, and potentially early EBs) and RB-specific genes such as *euo*, *incD*, and *CTL0238*-op, which we show were expressed only in the RB cell form.

The IB cell type is the transitional form between the RB and the EB and is currently poorly defined. We define the IB cell type as the committed step to EB formation; the IB is the cell form that exits the cell cycle and begins the program to transition into the infectious EB (Chiarelli et al., 2023; Appa Cody et al., 2024). Our data identified 67 genes that are likely expressed specifically in the IB cell type. The functions of these genes vary widely. We identified two porin genes (*porB* and *CLT0626*), two disulfide isomerases (*CTL0149* and *CTL0152*), and six polymorphic outer membrane proteins (*pmpB*, *C*, *E*, *F*, *G*, and *H*) as IB genes, suggesting dramatic changes to the outer membrane of the IB as it transitions into the EB.

The EB cell is the infectious cell form that is “terminally” differentiated. Once formed in the inclusion, the EB maintains an infectious phenotype through active metabolism but has very low levels of protein expression (Grieshaber et al., 2018). Here, we define the EB regulon as the genes expressed during the late IB-to-EB maturation phase. Of the 46 EB genes, 18 had been previously shown to be directly regulated by the sigma54 alternative sigma factor, and four were reported to be sigma28-regulated genes (Soules et al., 2020; Hatch Nathan and Ouellette Scot, 2023). The regulation of the remaining 24 genes is unknown. As both HctA ectopic expression and the ectopic expression of CtcB induce the expression of the EB genes, the EB regulon is likely regulated by a complex shift in gene expression, and the activation of the sigma54 and sigma28 regulons is a part of this shift.

We tested one of the predicted IB genes, *porB*, and showed that its regulation, both by promoter-specific gene expression in chlamydial cells and by its developmental kinetics, matched that of the IB gene *hctA*. We further confirmed this using FISH to demonstrate that cell type gene expression matched that of *hctA*. We have previously published the kinetics of the *euo*, *hctA*, and *hctB* promoters and showed that the promoter activities fit into the RB, IB, and EB models (Chiarelli et al., 2020; Chiarelli et al., 2023). Here, we combined these promoter-reporter strains with FISH and demonstrated that the *euo* mRNA was expressed primarily in RBs, that *hctA* mRNA was expressed in IBs, and that *hctB* mRNA was expressed in EBs, demonstrating the usefulness of FISH for identifying cell type-specific gene expression.

Overall, these data support a model that includes (at least) three dominant cell forms: the RB, the IB, and the EB. These cells have dramatically different gene expression profiles and phenotypes. The EB has been well characterized, as it is the infectious form, does not replicate, and has a dramatically condensed nucleoid. The nucleoid structure is due in part to the binding of the two histone-like proteins, HctA and HctB, to the chromosome (Barry et al., 1990; Brickman et al., 1991). Our data indicate that the construction of the compact nucleoid occurs in two distinct and temporally separated steps (Chiarelli et al., 2020; Chiarelli et al., 2023). HctA is expressed as an IB gene and, when ectopically expressed, results in the expression of the EB genes, suggesting that the HctA expression is an important regulator of the IB-to-EB transition. HctB, in contrast, is expressed as an EB gene and, when ectopically expressed, results in the inhibition of the expression of most genes with the exception of the ribosomal protein genes. An intriguing hypothesis is that the ribosomal protein genes are potentially free of inhibition in the mature EB, which could in turn allow protein synthesis to be rapidly reinitiated upon infection to aid in EB-to-RB differentiation, without a requirement for complete removal of HctA and HctB from the chromosome. Currently, the organization of the nucleoids and proteins that mediate compaction is an underexplored area of research. Taken together, these data suggest that the transition from the IB-to-EB occurs in two steps: 1) HctA chromosomal binding potentially turns off RB and IB genes, allowing EB genes to become expressed, and 2) HctB is expressed late in EB formation, creating the final condensed nucleoid and turning off the majority of gene expression but potentially sparing the ribosomal genes.

Volcano plots of the effects of the ectopic expression of the four regulatory genes support the categorization of most chlamydial genes into the RB, IB, and EB categories. The expression of the T3SS operons was specifically focused on, and it was observed that the majority of the operons for the structural components were IB-like in their regulation. This was verified using FISH for both the *sctJ* operon (*sctJ*, *sctK*, *sctL*, *sctR*, *sctS*, and *sctT*) and the *sctU* operon (*sctU*, *sctV*, *lcrD*, *copN*, *scc1*, and *malQ*). Additionally, the promoter for the *sctJ* operon was active in the IB cell form. While the majority of the T3SS structural operons were expressed as IB genes, the two translocon operons (*CTL0238*, *lcrH*, *copB_2*, and *copD_2*) and (s*cc2*, *CTL0840*, *copB*, and *copD*) were predicted by clustering and volcano plots to be expressed in RB and EB cells, respectively. This prediction was again verified by FISH in the context of dual promoter-reporter strains.

The observation that the two translocons were expressed in distinct cell forms (*CTL0238*-op in RBs and *scc2*-op in EBs) prompted us to determine the expression of the T3SS effectors. *Ctr* encodes two classes of effectors: soluble and inclusion membrane-embedded proteins (Incs) (Dehoux et al., 2011; Rockey et al., 2002; Lutter et al., 2012). The data from this study showed that the majority of the Inc protein effectors (28 out of 36) were expressed as RB genes while the majority of the soluble T3SS effectors (17 out of 23) were expressed as EB genes and that none of the soluble effectors were expressed as RB genes. This pattern supports an intriguing model: the *scc2*-op translocon translocates soluble effectors as the EB contacts host cells and mediates entry events, while *CTL0238*-op is expressed early during the EB-to-RB differentiation process in the nascent inclusion and translocates the transmembrane Inc effectors. Whether this separation is temporal or whether the two translocons are specialized for the translocation of soluble vs. inclusion membrane effectors is currently unknown. Interestingly, although the majority of the Inc proteins were expressed as RB genes, there were two Incs (*incV* and *incM*) that were determined to be expressed in EBs. In addition to their regulation pattern, we also verified that *incV* and *incM* were EB genes using FISH. Both *IncV* and *IncM* are involved in the establishment of early inclusion functions and are expressed late in the developmental cycle (Belland et al., 2003; Stanhope et al., 2017; Luís et al., 2023). We hypothesize that these “pre-loaded” Inc proteins are among the first to be secreted from internalized *Ctr* after *CTL0238*-op is deployed.

*Ctr* communicates and reprograms the host cell to create and maintain its intracellular replication niche in part through the use of the T3SS. We were surprised that the majority of the T3SS operons for the structural components of the system were expressed as IB genes. This expression pattern along with the cell type-specific expression of the translocons (one in the RB and one in the EB) and effectors suggests that the T3SS is constructed, is deployed, and secretes effectors in a cell type-specific manner that is likely a critical component of the complex developmental cycle and host cell reprogramming.

**Figure 11:**
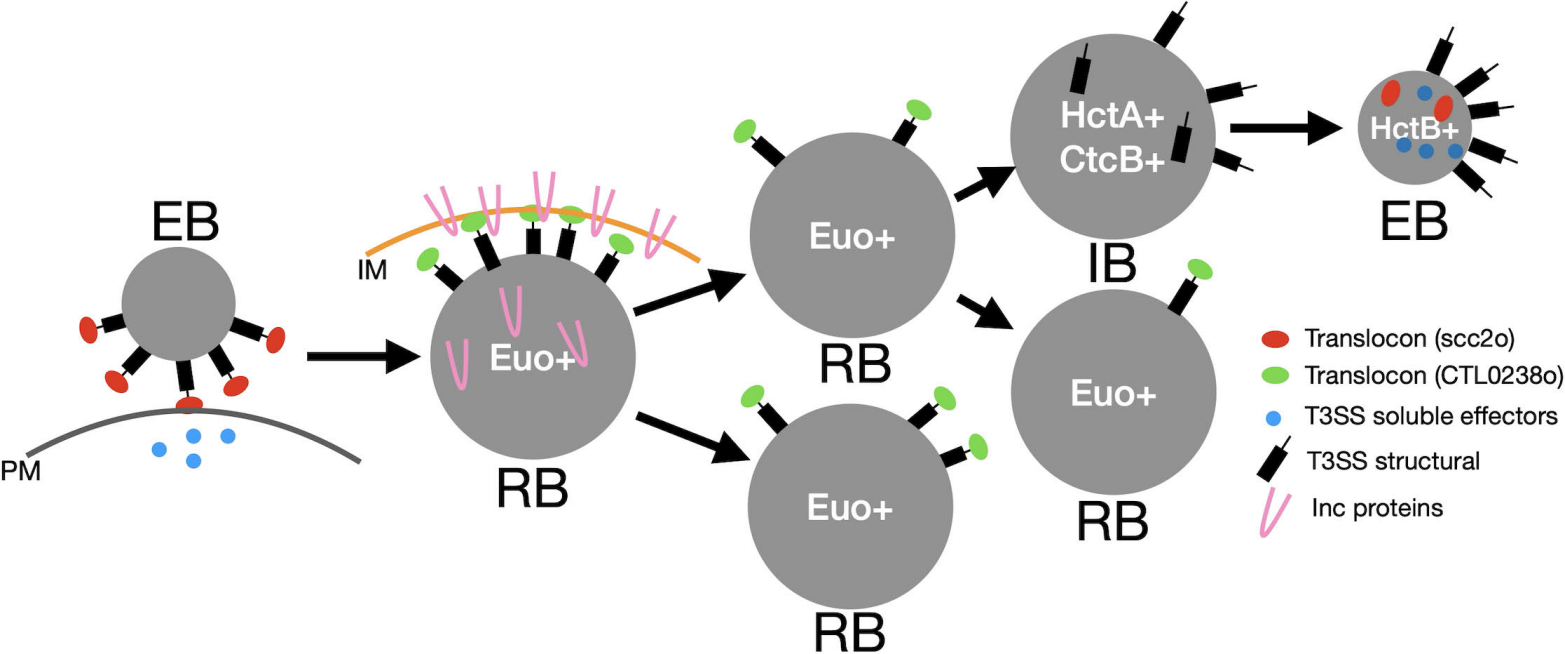
Model of cell type-specific deployment of the T3SS. In this model, the *scc2-*op translocon secretes effectors across the plasma membrane (PM) for host cell entry. The *scc2*-op translocon is replaced in the RB with the *CTL0239-*op translocon for the secretion of the Inc proteins across the inclusion membrane (IM). The structural components of the T3SS are then reconstructed during the IB-to-EB maturation phase. T3SS, type III secretion system; RB, reticulate body; IB, intermediate body; EB, elementary body.

Our model depicted in **Figure 11** suggests that the EB binds to and enters cells in part through the deployment of soluble effectors and the *scc2*-op translocon expressed during EB development. After entry, EB-to-RB differentiation begins, and the RB genes are expressed; this includes the *CTL0238*-op translocon, which deploys the Inc proteins for the creation of the inclusion replication niche and the genes required for chlamydial replication, leading to RB amplification. After an amplification period, the RB matures into a stem cell-like cell form and begins to produce IBs (Chiarelli et al., 2023). The T3SS structural components are assembled in the IB, and this facilitates maturation to the EB form (Chiarelli et al., 2020; Chiarelli et al., 2023). That the IB and not the RB expresses the genes for the construction of the T3SS suggests that the T3SS apparatus deployed on the EB cells remains on the RBs and is diluted with every round of replication. It is unclear if the secretion system is partitioned equally or is retained in a subset of RBs​​. Intriguingly, this supports a proposed role of T3SS dilution in cell form maturation/development put forth previously (Peters et al., 2005; Hoare et al., 2006; Wilson et al., 2006).

The IB also expresses the histone-like DNA-binding protein, HctA. Previous studies have shown that when expressed in *E. coli*, HctA can alter gene expression in a gene-specific manner (Barry et al., 1990). Our data suggest that HctA has an important role in shifting gene expression from the IB pattern to the EB genes. This is likely in conjunction with the CtcB/C two-component regulatory system and sigma54 (Soules et al., 2020; Hatch Nathan and Ouellette Scot, 2023). The EB genes, as previously mentioned, include the majority of the soluble T3SS effectors and the *scc2*-op translocon as well as the HctB DNA-binding protein. We hypothesize that EB gene expression loads the EB with the invasion-related proteins and that HctB shuts down the majority of gene expression, creating the final condensed nucleoid, the final step of EB formation. This prepares the EB for the initiation of the next round of infection (**Figure 11**).

DNA replication is tightly controlled during the *Ctr* developmental cycle; only the RB cell form replicates the chromosome, and the IB and EB cells contain a single fully replicated chromosome (Grieshaber et al., 2021; Appa Cody et al., 2024). The role of the control of DNA replication in regulating gene expression is currently unknown. However, it is intriguing to speculate that DNA replication could contribute to changes in DNA supercoiling, which has been shown to play a role in gene expression during the chlamydial developmental cycle (Niehus et al., 2008; Cheng and Tan, 2012; Shen et al., 2024).

Our data have highlighted three categories of gene expression that define the three major phenotypic cell forms: RB, IB, and EB. However, future studies are needed to define the regulatory circuits and DNA elements that create these cell form-specific expression patterns. The identification of cell type gene expression of a large percentage of the chlamydial genome will aid the determination of the functions of the many hypothetical genes encoded in the chlamydial genome. Understanding the function of many of these genes has been hampered by the mixed-cell environment of chlamydial inclusion. Additionally, with the emerging genetic tools available to investigate the functional roles of genes during infection, knowing in which cell type a gene is expressed will improve the interpretation of the data.

## Materials and methods

### Cell culture

Cell lines were obtained from the American Type Culture Collection. Cos-7 cells (CRL-1651) were grown in RPMI-1640 and supplemented with 10% fetal bovine serum (FBS) and 10 μg/mL gentamicin (Cellgro). *C. trachomatis* serovar L2 (LGV Bu434) was grown in Cos-7 cells. EBs were purified by density gradient (DG) centrifugation essentially as described (Howard et al., 1970) following 48 hours of infection. EBs were stored at −80°C in Sucrose Phosphate Glutamate (SPG) buffer [10 mM sodium phosphate (8 mM K_2_HPO_4_ and 2 mM KH_2_PO_4_], 220 mM sucrose, and 0.50 mM l-glutamic acid, pH 7.4] until use.

### Vector construction

All constructs used p2TK2-SW2 (Derré et al., 2011) as the backbone, and cloning was performed using the In-fusion HD EcoDry Cloning kit (Thermo Fisher Scientific, Waltham, MA, USA). Primers and geneblocks (gBlocks) were ordered from Integrated DNA Technologies (IDT, Coralville, IA, USA) and are noted in **Supplementary Table S6**. For the ectopic expression of Clover, Euo, CtcB, and HctA, the T5 promoter (*E. coli* sigma70 constitutive promoter) and the E riboswitch were used for conditional translational expression control using the inducer, theophylline (Tph) (Grieshaber et al., 2022). For the ectopic expression of HctB, the Tet promoter was used in conjunction with the E riboswitch to confer both transcriptional and translational expression controls (Tet-JE-hctB) and has been described previously (Grieshaber et al., 2022). The *hctA*, *hctB*, *euo*, and *ctcB* ORFs were amplified from *Ctr* L2(434) using the primers indicated in **Supplementary Table S6**.

To create the Scarlet-I reporters *hctB*prom_Scarlet-*euo*prom_neongreen (BsciEng), *hctA*prom_Scarlet-*euo*prom_neongreen (AsciEng), *porB*prom_Scarlet-*euo*prom_neongreen (PsciEng), and *sctJ*prom_Scarlet-*euo*prom_neongreen (JsciEng), the gBlock mScartlet-I (**Supplementary Table S6**) was cloned into BMELVA (Chiarelli et al., 2023) to replace the mKate RFP gene. The degradation tag LVA was then removed from the Neongreen using the primers indicated. The *hctA*, *porB*, and *sctJ* promoters were amplified and used to replace the *hctB* promoter using the primers indicated to create AsciEng, PsciEng, and JsciEng, respectively.

#### Chlamydial transformation and isolation

The transformation of Ctr L2 was performed essentially as previously described (Chiarelli et al., 2020). Briefly, 1 × 10^8^ EBs + >2 µg DNA/well were used to infect a 6-well plate. Transformants were selected over successive passages with 1 U/mL penicillin G or 500 µg/mL spectinomycin as appropriate for each plasmid. The new strain was clonally isolated via successive rounds of inclusion isolation (MOI < 1) using a micromanipulator. The clonality of each strain was confirmed by isolating the plasmid, transforming it into *E. coli*, and sequencing six transformants.

The chlamydial strains L2-E-euo-FLAG, L2-E-hctA-FLAG, and L2-E-ctcB-FLAG were induced at the indicated times with 0.5 mM Tph. As described previously, *Ctr* could not successfully be transformed with the E-hctB-FLAG construct; therefore, we developed a tet-riboJ-E promoter system that combines both transcriptional and translational controls to hctB-FLAG expression, creating the strain L2-tet-J-E-hctB-FLAG (Grieshaber et al., 2022). The expression of HctB-FLAG was induced with 0.5 mM Tph + 30 ng/mL anhydrotetracycline (aTc).

#### Replating assay

*Ctr* were isolated by scraping the infected monolayer into media and pelleting at 17,200 rcfs. The EB pellets were resuspended in RPMI via sonication and seeded onto fresh monolayers in a 96-well microplate in a twofold dilution series. Infected plates were incubated for 24 hours prior to fixation with methanol and stained with 4′,6-diamidino-2-phenylindole (DAPI) and *Ctr* MOMP Polyclonal Antibody, FITC (Thermo Fisher Scientific). The DAPI stain was used for automated microscope focus and visualization of host-cell nuclei, and the anti-*Ctr* antibody was used to visualize the *Ctr* to identify and count inclusions. Inclusions were imaged using a Nikon Eclipse TE300 inverted microscope utilizing a scopeLED lamp at 470 and 390 nm and BrightLine band pass emissions filters at 514/30 nm and 434/17 nm. Image acquisition was performed using an Andor Zyla sCMOS in conjunction with the μManager software. Images were analyzed using the ImageJ software and custom scripts. Statistical comparisons between treatments were performed using an ANOVA test followed by Tukey’s honestly significant difference test.

### Transmission electron microscopy

For the analysis of the structure of *Ctr* upon ectopic protein expression, cell monolayers were infected with the indicated strain at an MOI of 0.5 and induced with 0.5 mM Tph for the E riboswitch constructs and with 0.5 mM Tph and 30 ng/mL aTc for the tet-E-J promoter constructs. All inductions were performed at 15 hpi. Infected cells were released from the plate with Trypsin-EDTA at 30 hpi and rinsed with 1× phosphate buffered saline (PBS), and the pellet was fixed with EM fixative [% paraformaldehyde (PFA), 2% glutaraldehyde, and 0.1 M phosphate buffer, pH 7.2] overnight at 4°C. Fixed pellets were rinsed and dehydrated before embedding with Spurr’s resin and cross sectioned using an ultramicrotome (Riechert Ultracut R; Leica, Wetzlar, Germany). Ultra-thin sections were placed on formvar coated slot grids and stained with uranyl acetate and Reynolds lead citrate. TEM imaging was conducted using a Tecnai G2 transmission electron microscope (FEI Company, Hillsboro, OR, USA).

### RNA-seq

The expression of each protein was induced at 15 hpi with either 0.5 mM Tph and 30 ng/mL anhydrotetracycline (HctB) or 0.5 mM Tph (Clover, HctA, CtcB, and Euo) and the *Ctr* isolated at 18 and 24 hpi on ice. Total RNA was isolated from the indicated infections and treatments. Briefly, the infected monolayer was scraped into ice-cold PBS and lysed using a Dounce homogenizer, and the *Ctr* was isolated over a 30% MD-76R pad. Total RNA was isolated using TRIzol reagent (Life Technologies, Carlsbad, CA, USA) following the protocol provided, and genomic DNA was removed (TURBO DNA-free Kit, Invitrogen, Carlsbad, CA, USA). Both prokaryotic and eukaryotic rRNAs were depleted using Illumina Ribo-Zero Plus. The enriched RNA samples were quantified, and the libraries were built and barcoded by the IBEST Genomics Resources Core at the University of Idaho. The libraries were sequenced by the University of Oregon sequencing core using the Illumina NovaSeq platform. The chlamydial reads were analyzed by aligning to the published *Ctr* L2 Bu 434 genome using the Bowtie2 aligner software (Langmead and Salzberg, 2012). The aligned chlamydial reads were quantified for each chlamydial ORF using HTseq. For each sample, ~1 × 10^6^ read pairs were counted for 904 chlamydial ORFs resulting in approximately 1,000× coverage for each ORF. Statistical analysis and normalization of read counts were accomplished using DESeq2 in R (Love et al., 2012). Log2fold change and statistics were also calculated using DESeq2. Heatmaps and hierarchical clustering were generated and visualized using Python with Pandas and the Seaborn visualization package (Waskom, 2021). The raw reads and HT-seq counts are accessible from the NCBI’s Gene Expression Omnibus with the accession number GSE287626. Volcano plots were constructed from the log2fold change data using Python and the Bokeh plotting library (Bokeh Development Team).

### RNA fluorescence *in situ* hybridization

All FISH probes were designed by Molecular Instruments (Los Angeles, CA, USA) using the sequence indicated in (**Supplementary Table S7**). Cos-7 monolayers seeded on coverslips were infected with the indicated strains at an MOI ~ 0.3. Infected cells were fixed at the indicated times in 4% PFA for 10 min at room temperature (RT) at 24 hpi, washed 2× with 1× PBS, and dehydrated overnight at −20°C in 70% EtOH. Samples were probed, and the signal was amplified as described by the protocol provided by Molecular Instruments with the exception that DAPI was added to the final wash to visualize DNA. Coverslips were mounted on a microscope slide with MOWIOL^®^ mounting solution (100 mg/mL MOWIOL^®^ 4-88, 25% glycerol, and 0.1 M Tris, pH 8.5).

Fluorescence images were acquired using a Nikon spinning disk confocal system with a 60× oil-immersion objective, equipped with an Andor Ixon EMCCD camera, under the control of the Nikon Elements software. The imaged fields of view were captured at random by generating a grid pattern and automated imaging. Images were processed using the image analysis software ImageJ (http://rsb.info.nih.gov/ij/). Representative confocal micrographs displayed in the figures are maximal intensity projections of the 3D data sets, unless otherwise noted.

### Chlamydial cell mRNA expression quantification

Individual chlamydial cells from 3D confocal images of individual inclusions were identified using the ImageJ plugin TrackMate (Tinevez et al., 2014). An example of the results of the TrackMate cell identification output is shown in **Supplementary Figure S5**. Briefly, each cell was detected in multiple slices and connected to form a track. The center slice for each track was considered the center of each cell, and the fluorescence intensity for each channel was determined for each detected cell. The chlamydial cells were expressing cell form-specific markers, so for each inclusion, cells were detected for each marker (neongreen or scarlet-I). Fields of view to collect inclusion images were selected at random, and five inclusions were randomly selected from the data sets. The data were plotted using python.

### Live cell imaging

Monolayers of Cos-7 cells were grown in glass-bottom 24-well plates and infected with the promoter-reporter strains L2-BsciEng, L2-AsciEng, and L2-PsciEng. Live cell imaging of the developing inclusions was started at 8 hpi using an automated Nikon epifluorescent microscope equipped with an Okolab (http://www.oko-lab.com/live-cell-imaging) temperature-controlled stage and an Andor Zyla sCMOS camera (http://www.andor.com). Multiple fields of view from each well were imaged every 15 minutes. The fluorescence intensity of each inclusion over time was tracked using the ImageJ plugin TrackMate (Tinevez et al., 2014), and the results were averaged and plotted using python and matplotlib (Shukla and Parmar, 2014).

## Data Availability

The datasets presented in this study can be found in online repositories. The names of the repository/repositories and accession number(s) can be found below: https://www.ncbi.nlm.nih.gov/geo/, GSE287626.

## Supplementary material

Supplementary Figure 1 Western analysis of ectopically expressed Clover, Euo, HctA, CtcB and HctB. To ensure the FLAG constructs expressed protein of the correct size, infected and induced monolayers were lysed in reducing lane marker sample buffer and protein lysates were separated on 10% SDS-PAGE gels and transferred to a nitrocellulose membrane for western analysis of the FLAG-tagged protein. The membrane was blocked with PBS + 0.1% Tween 20 (PBS-T) and 5% nonfat milk prior to incubating in monoclonal anti-FLAG M2 antibody (1:40,000, Sigma, Thermo Scientific™) overnight at 4 °C followed by goat-anti mouse IgG-HRP secondary antibody (Invitrogen™) at room temperature for 2 hours. The membrane was developed with the Supersignal West Dura luminol and peroxide solution (Thermo Scientific™) and imaged using an Amersham Imager 600. Predicted sizes are as follows: Clover-FLAG (29 kDa), HctB-FLAG (26.6 kDa), Euo-FLAG (23.7 kDa), HctA-FLAG (16.5 kDa), CtcB-FLAG (42.5 kDa).

Supplementary Figure 2 Cell type specific activity of the *porB* promoter. Cos-7 cells infected with the strain L2-PsciEng expressing Neongreen from the *euo* promoter (green) and Scarlet-I from the *porB* promoter (magenta). At 16 hpi there was only a single *porB* positive cell detected (arrow) while the rest of the chlamydial cells were only euoprom+. At 24 hpi there were two distinct cell populations, *euo*prom+ (green) and *porB*prom+ (magenta) cells. Size bar = 5µm.

Supplementary Figure 3 IB cell type expression of the T3SS structural operon *sctU*-op. (A) Cells were infected with L2-AsciEng for 16 hpi and fixed and stained using a FISH probe (*sctU* through *lcrD)* to the mRNA for the T3SS structural operon sctU-op. All cells were positive for *euo*prom expression (green) and negative for *sctU-op* mRNA (magenta). Infected cells were also probed for *hctA* mRNA expression and *euo* mRNA. Like *sctU*o the cells had little signal for the *hctA* mRNA. However, the *euo*prom+ cells were also positive for the *euo* mRNA (B) Cells were infected with L2-AsciEng and L2-BsciEng for 24 hpi and fixed and stained using FISH for the *sctU*-op mRNA. For the *euo*prom sample, the *sctU*-op FISH signal (magenta) was present in a distinct subset of cells and not in the majority of the *euo*prom+ cells (green). TrackMate was used to identify the *sctU*-op mRNA+ cells and the signal for *euo*prom and FISH were quantified for each *sctU*-op+ cell. The converse was also performed, the *euo*prom+ cells were identified (green) and the *euo*prom signal and FISH signal was quantified for each *euo*prom+ cell. The fluorescence intensity for each channel for both cell populations was plotted. The FISH signal was also compared to the *hctA*prom expression pattern and showed subsets of cells that were stained for both *sctU*-op mRNA and *hctA*prom expression as well as non overlapping populations. The *sctU*-op mRNA+ cells were again identified using TrackMate (magenta) and the signal for *hctA*prom and FISH were quantified for each *sctU*-op+ cell. Each *hctA*prom+ cell was also identified (green) and the FISH and *hctA*prom signal was determined and plotted for both cell populations. The *sctU-*op FISH staining was also compared to the expression from the *hctB*prom reporter. The *sctU*-op mRNA FISH staining was again present in a subset of cells but showed little overlap with the *hctB*prom fluorescent signal. The FISH signal and *hctB*prom signal were measured in both cell populations (*sctU* mRNA+ cells (magenta) and *hctB*prom+ cells (green) and plotted. Both populations were primary single positive, either *sctU*-op mRNA high or *hctB*rpom high but rarely both. Size bar = 5µm.

Supplementary Figure 4 Cell type expression of *incM*. Cos-7 cells infected with L2-BsciEng for 16 and 24 hpi and stained for *incM* mRNA expression using custom FISH probes. The *incM* mRNA signal (magenta) was undetected at 16 hpi. At 24 hpi the *incM* mRNA signal showed overlap with the *hctB*prom signal (yellow) but not the *euo*prom signal (green). Size bar = 5µm.

Supplementary Figure 5 Example image of individual *Chlamydia* detected using Trackmate. Pictured is a single Image slice of an inclusion from a cell monolayer infected with L2-PsciEng and fixed at 24 hpi. The inclusion image was captured using confocal microscopy and the 3d image stack was used to identify each cell within the inclusion. The pink circles identify each cell in each slice and the yellow squiggles track each cell through the 3d stack. The central point of each cell is identified by the middle of the track.

Supplementary Table 1 Chlamydial genes designated as RB, IB, EB expressed genes and their regulation from ectopic expression of Euo, HctA, CtcB and HctB.

Supplementary Table 2 T3SS structural operons and cell form expression.

Supplementary Table 3 T3SS effector gene regulation from ectopic expression of Euo, HctA, CtcB and HctB.

Supplementary Table 4 Inc gene regulation from ectopic expression of Euo, HctA, CtcB and HctB.

Supplementary Table 5 List of primers used to construct plasmids.

Supplementary Table 6 List of FISH probes and the location on the *Ctr* genome.
