# Supplementary figures and images for: The T3SS structural and effector genes of Chlamydia trachomatis are expressed in distinct phenotypic cell forms
Source: Front Cell Infect Microbiol. 2025 May 8;15:1579247. doi: 10.3389/fcimb.2025.1579247 (PMC12095286; doi:10.3389/fcimb.2025.1579247)

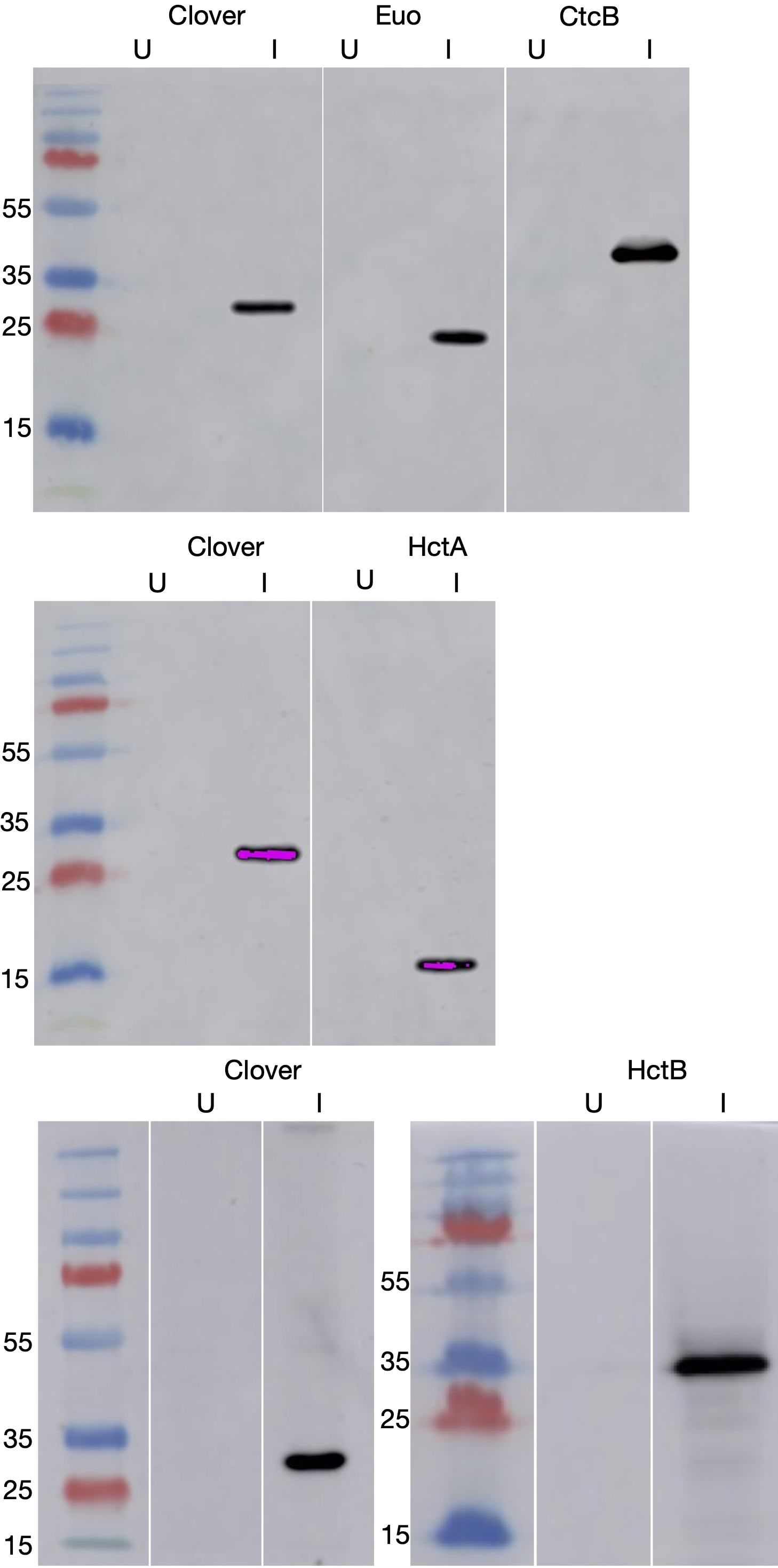

Supplement: Supplementary Figure 1 — Western analysis of ectopically expressed Clover, Euo, HctA, CtcB and HctB. To ensure the FLAG constructs expressed protein of the correct size, infected and induced monolayers were lysed in reducing lane marker sample buffer and protein lysates were separated on 10% SDS-PAGE gels and transferred to a nitrocellulose membrane for western analysis of the FLAG-tagged protein. The membrane was blocked with PBS + 0.1% Tween 20 (PBS-T) and 5% nonfat milk prior to incubating in monoclonal anti-FLAG M2 antibody (1:40,000, Sigma, Thermo Scientific™) overnight at 4 °C followed by goat-anti mouse IgG-HRP secondary antibody (Invitrogen™) at room temperature for 2 hours. The membrane was developed with the Supersignal West Dura luminol and peroxide solution (Thermo Scientific™) and imaged using an Amersham Imager 600. Predicted sizes are as follows: Clover-FLAG (29 kDa), HctB-FLAG (26.6 kDa), Euo-FLAG (23.7 kDa), HctA-FLAG (16.5 kDa), CtcB-FLAG (42.5 kDa). [file Image1.tiff]

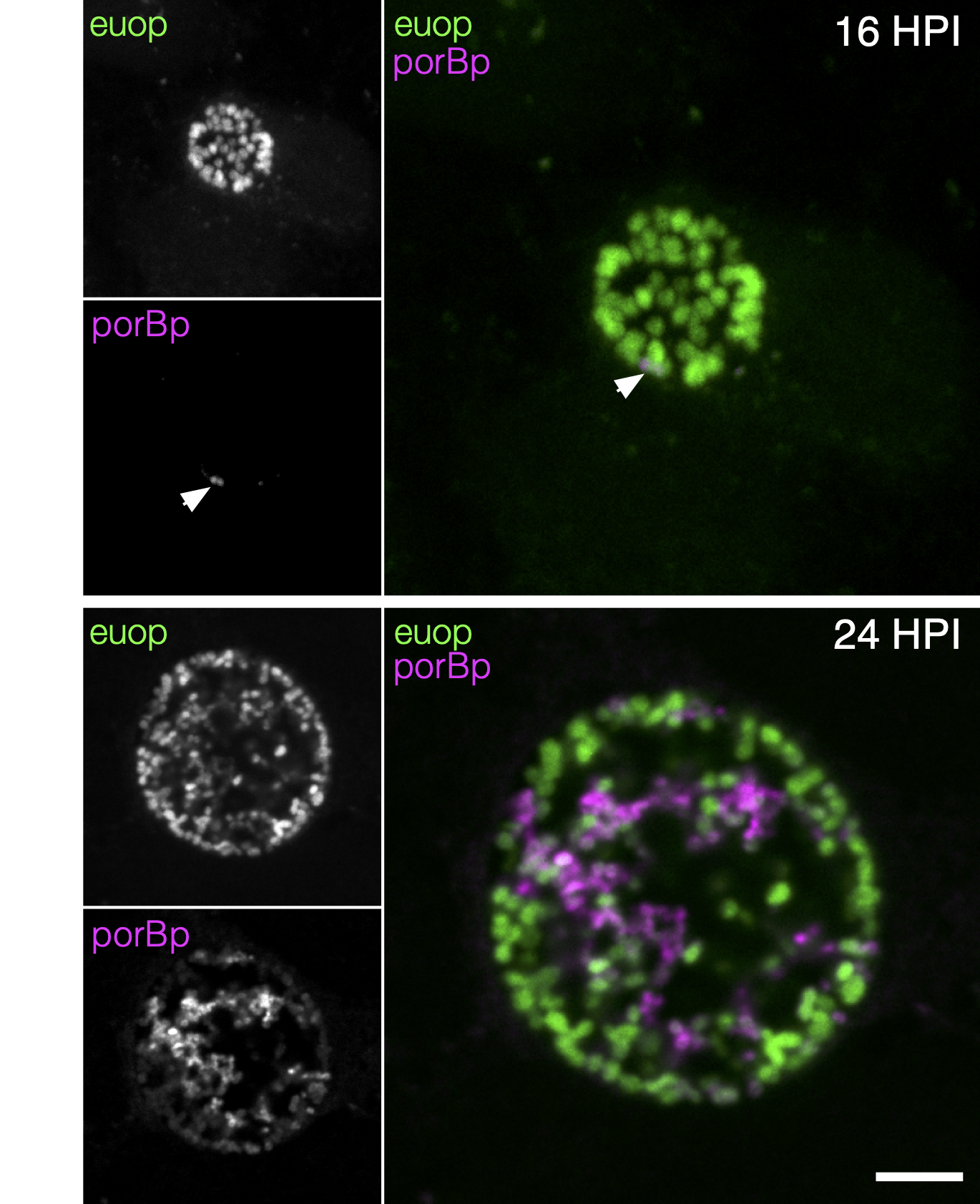

Supplement: Supplementary Figure 2 — Cell type specific activity of the porB promoter. Cos-7 cells infected with the strain L2-PsciEng expressing Neongreen from the euo promoter (green) and Scarlet-I from the porB promoter (magenta). At 16 hpi there was only a single porB positive cell detected (arrow) while the rest of the chlamydial cells were only euoprom+. At 24 hpi there were two distinct cell populations, euoprom+ (green) and porBprom+ (magenta) cells. Size bar = 5µm. [file Image2.tiff]

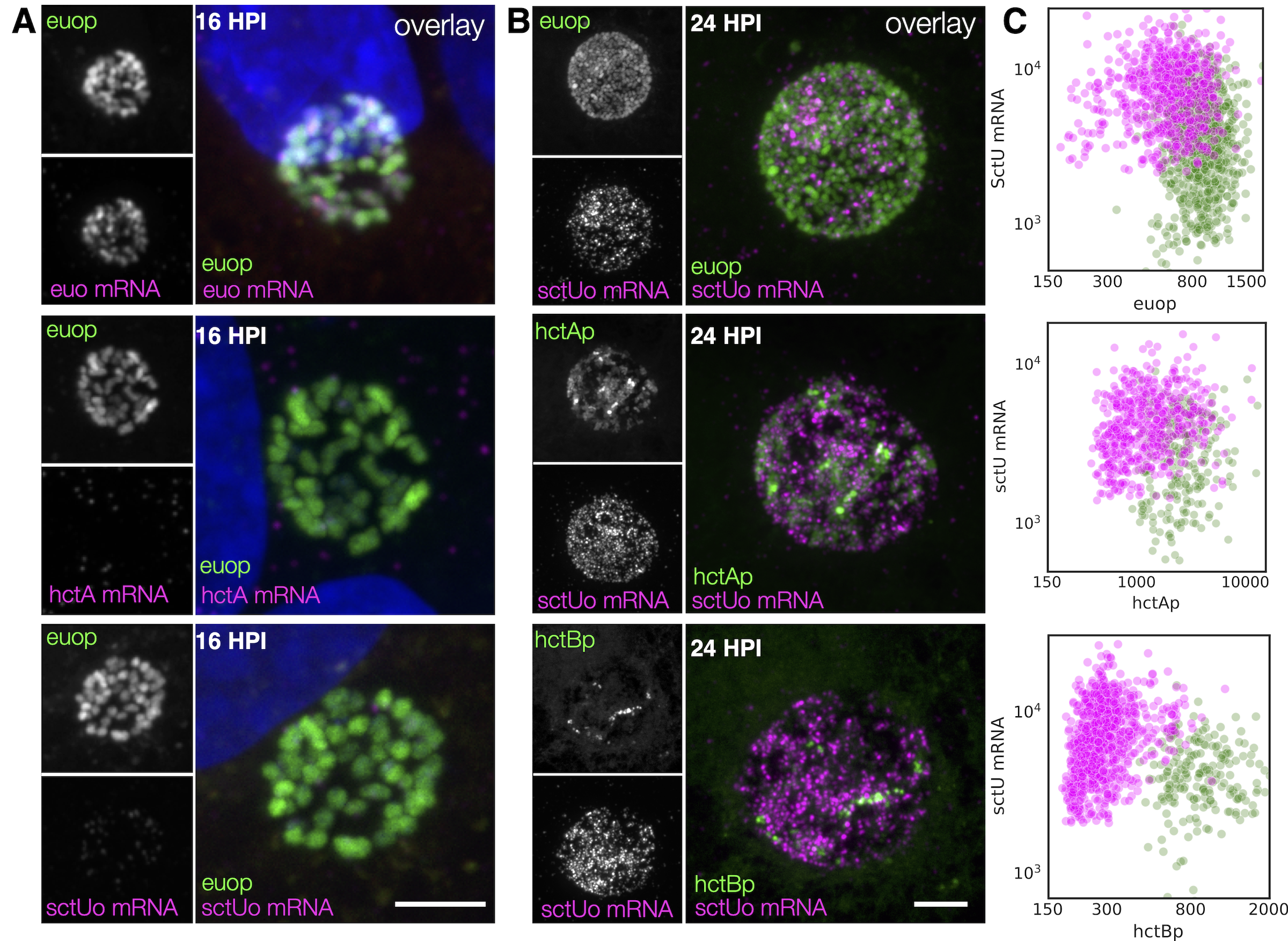

Supplement: Supplementary Figure 3 — IB cell type expression of the T3SS structural operon sctU-op. (A) Cells were infected with L2-AsciEng for 16 hpi and fixed and stained using a FISH probe (sctU through lcrD) to the mRNA for the T3SS structural operon sctU-op. All cells were positive for euoprom expression (green) and negative for sctU-op mRNA (magenta). Infected cells were also probed for hctA mRNA expression and euo mRNA. Like sctUo the cells had little signal for the hctA mRNA. However, the euoprom+ cells were also positive for the euo mRNA (B) Cells were infected with L2-AsciEng and L2-BsciEng for 24 hpi and fixed and stained using FISH for the sctU-op mRNA. For the euoprom sample, the sctU-op FISH signal (magenta) was present in a distinct subset of cells and not in the majority of the euoprom+ cells (green). TrackMate was used to identify the sctU-op mRNA+ cells and the signal for euoprom and FISH were quantified for each sctU-op+ cell. The converse was also performed, the euoprom+ cells were identified (green) and the euoprom signal and FISH signal was quantified for each euoprom+ cell. The fluorescence intensity for each channel for both cell populations was plotted. The FISH signal was also compared to the hctAprom expression pattern and showed subsets of cells that were stained for both sctU-op mRNA and hctAprom expression as well as non overlapping populations. The sctU-op mRNA+ cells were again identified using TrackMate (magenta) and the signal for hctAprom and FISH were quantified for each sctU-op+ cell. Each hctAprom+ cell was also identified (green) and the FISH and hctAprom signal was determined and plotted for both cell populations. The sctU-op FISH staining was also compared to the expression from the hctBprom reporter. The sctU-op mRNA FISH staining was again present in a subset of cells but showed little overlap with the hctBprom fluorescent signal. The FISH signal and hctBprom signal were measured in both cell populations (sctU mRNA+ cells (magenta) and hctBprom+ cells (green) [file Image3.tiff]

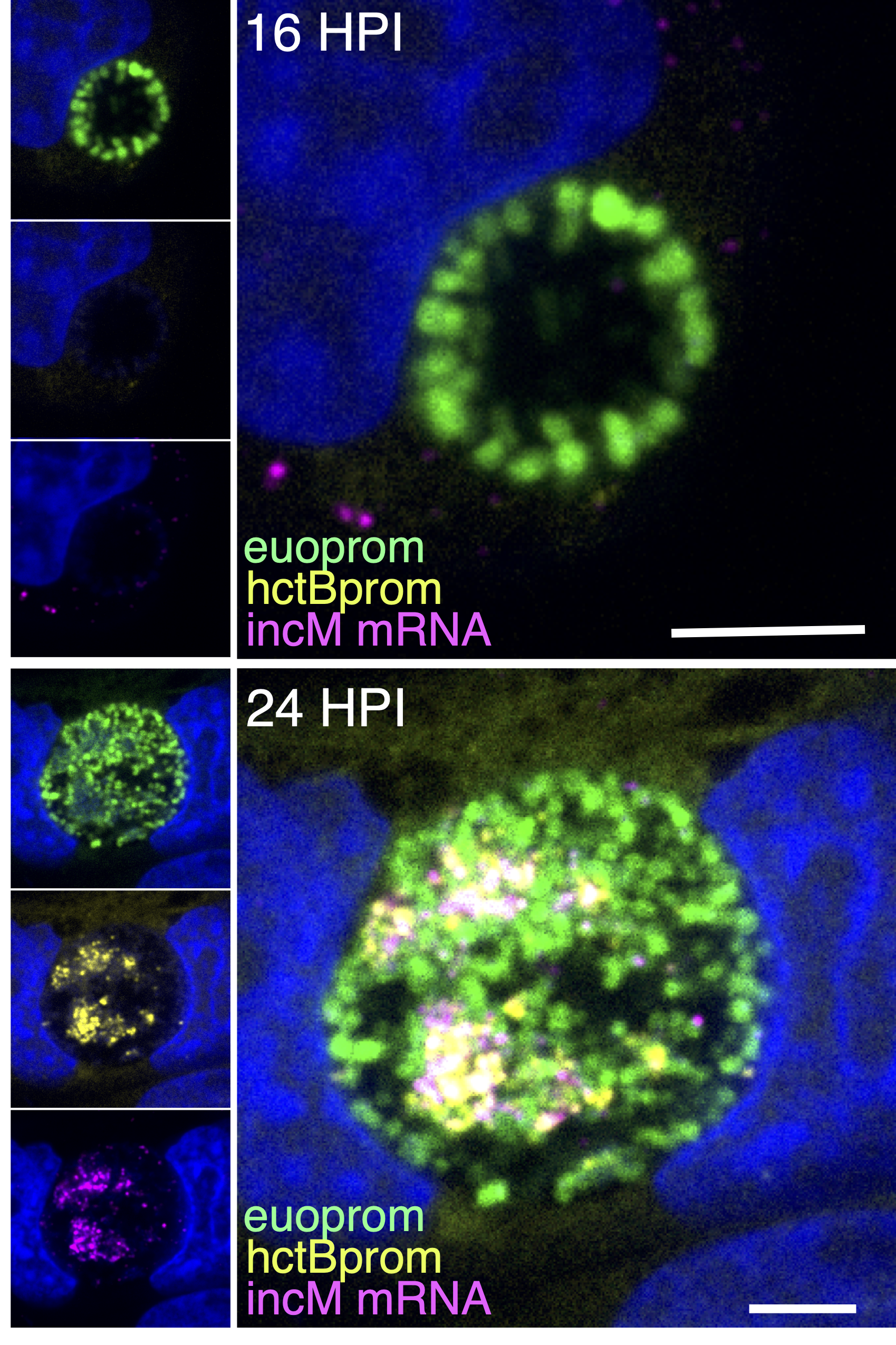

Supplement: Supplementary Figure 4 — Cell type expression of incM. Cos-7 cells infected with L2-BsciEng for 16 and 24 hpi and stained for incM mRNA expression using custom FISH probes. The incM mRNA signal (magenta) was undetected at 16 hpi. At 24 hpi the incM mRNA signal showed overlap with the hctBprom signal (yellow) but not the euoprom signal (green). Size bar = 5µm. [file Image4.tiff]

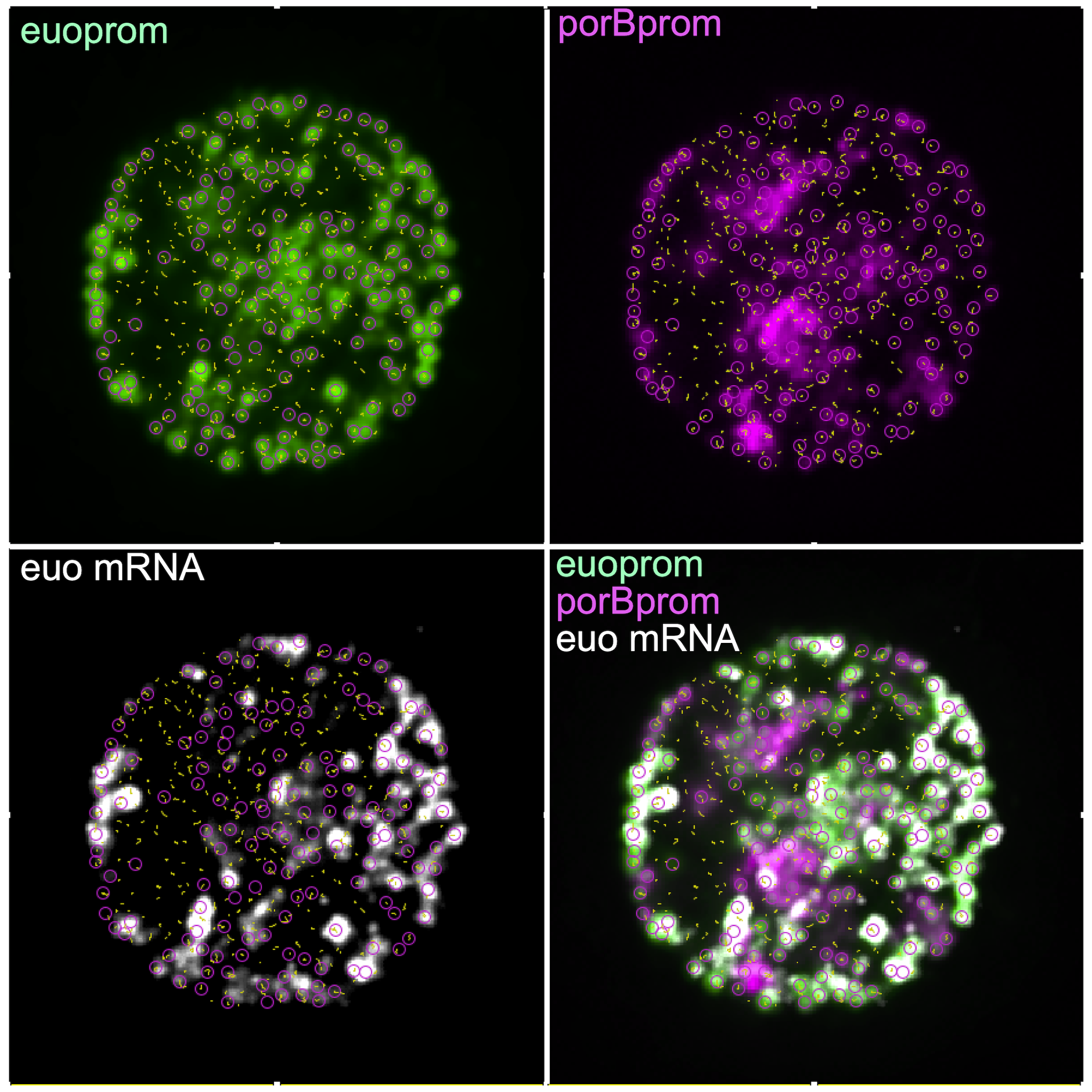

Supplement: Supplementary Figure 5 — Example image of individual Chlamydia detected using Trackmate. Pictured is a single Image slice of an inclusion from a cell monolayer infected with L2-PsciEng and fixed at 24 hpi. The inclusion image was captured using confocal microscopy and the 3d image stack was used to identify each cell within the inclusion. The pink circles identify each cell in each slice and the yellow squiggles track each cell through the 3d stack. The central point of each cell is identified by the middle of the track. [file Image5.tiff]
